# *Casimiroa edulis* Leaf Extract–Loaded PLGA Nanoparticles: Untargeted Phytochemical Profiling and Wound-Healing-Oriented Antioxidant/Occlusive Characterization

**DOI:** 10.3390/pharmaceutics18020249

**Published:** 2026-02-17

**Authors:** Clara Luisa Domínguez-Delgado, Mariana Montserrat Guadarrama-López, Yair Cruz-Narváez, Rafael Iván Puente-Lee, Sergio Arturo Ojeda-Piedra, María de la Luz Zambrano-Zaragoza

**Affiliations:** 1 Laboratorio de Procesos de Transformación y Tecnologías Emergentes de Alimentos-UIM, Facultad de Estudios Superiores Cuautitlán-Campo 4, Universidad Nacional Autónoma de México, Km 2.5 Carretera Cuautitlán–Teoloyucan, San Sebastián Xhala, Cuautitlán Izcalli, Mexico State 54714, Mexico; mariana.montse06@gmail.com (M.M.G.-L.); sergioaop@comunidad.unam.mx (S.A.O.-P.); 2 Laboratorio de Posgrado e Investigación de Operaciones Unitarias, Escuela Superior de Ingeniería Química e Industrias Extractivas, Instituto Politécnico Nacional, Ciudad de México 07738, Mexico; ycruzn@ipn.mx; 3 Laboratorio de Microscopía Electrónica, Facultad de Química, Universidad Nacional Autónoma de México, Edificio H Mario Molina, Circuito Mario de la Cueva, Esquina Circuito de la Investigación Científica, Ciudad Universitaria, Coyoacán, Mexico City 04510, Mexico; ipl@unam.mx

**Keywords:** PLGA, polymeric nanoparticles, rapid emulsion-diffusion method, *Casimiroa edulis*, wound healing, skin dressings

## Abstract

**Background:** Nanoparticles are a promise for wound-healing therapies. However, its lack of efficacy/safety represents a real challenge for therapeutic use. **Objectives:** To overcome these problems, the ethanolic extract of *Casimiroa edulis* leaves, previously reported for its anti-inflammatory, antibiotic, and antioxidant activities, was characterized by FIA-ESI-FTICR-MS and encapsulated in biodegradable nanoparticles for potential wound-healing therapies. **Methods:** *Casimiroa edulis*-loaded nanoparticles (CE-NP) were prepared using the rapid emulsion-diffusion method and characterized by their particle size distribution, molecular interactions, charge, morphology, pH, physical stability, and antioxidant and occlusive effects. **Results:** A total of 40/34 ions in positive/negative electrospray ionization modes were obtained from the extract exploration analysis and were putatively annotated by accurate mass against databases with an error tolerance ≤10 mDa. The most abundant compounds showed the following order: tetramethylscutellarein > rutin > S-usnate > lactose > eugenol derivative > rotenone. While polyphenols predominated, carbohydrates, depsidones/other phenolics, etc., were also detected. The solid/spherical nanoparticles observed by TEM were obtained with a blend of acetone:methyl ethyl ketone (75:25) as the organic phase, producing a unimodal particle size (169.30 ± 1.30 nm; PdI = 0.08 ± 0.03). The encapsulation/loading percentages were 57 ± 0.74/1.62 ± 0.02%, ensuring an entrapment of half the extract, as observed in the FTIR studies. The light backscatter profiles show minimal differences, indicating physical stability correlated with the Z potential (−9.45 ± 1.73 mV). The antioxidant activity of the extract/nanoparticles at 40 µg/mL was 17.27 ± 2.86/16.73 ± 1.28 μg/mL, two-fold higher than that previously reported for sapote seeds. **Conclusions:** Biodegradable CE-NP with suitable characteristics were obtained for the first time, representing a preliminary proposal for wound healing. Efficacy studies are required.

## 1. Introduction

Recently, enhanced and multifunctional properties have been reported for wound-healing therapies using emerging nanoparticle-based technologies [1]. However, problems with efficacy, microbial infection, or antibiotic resistance [2], as well as toxicity (cytotoxicity and tissue accumulation), especially with metallic nanoparticles like silver (AgNPs) and zinc oxide (ZnONPs) [3], poor biodegradability and stability are still present in nanotherapies [4]. Natural compounds from plant extracts, such as *Casimiroa edulis,* with antioxidant [5], anti-inflammatory [6], antimicrobial [7], and anticoagulant [8] activities, as well as a mitigator of toxicity induced by aluminum [9] and silver nanoparticles [10], can provide a solution to improve the wound-healing process and reduce scar formation [11]. *Casimiroa edulis* encapsulated into biodegradable nanoparticles could enhance its therapeutic properties, overcoming the nanotherapies’ problems mentioned before, offering a good alternative for millions of patients globally.

Wound-healing management is associated with costs of billions of dollars each year [12], requiring treatment annually for millions of patients worldwide, leading to increased hospital stays, frequent medical interventions, and increased health care costs. Treatments are usually focused on reducing pain, mobility, and the risk of infection [13]. In the worst cases, patients with acute and chronic wounds may suffer long-term disability or even die if therapy is unsuccessful. Regarding the use of nanomaterials, they provide opportunities for improving wound healing, offering therapeutic advantages in various procedures. Nanoparticles can also protect active compounds from the body’s physical and chemical changes (pH, ionic strength, enzymatic activity, etc.), preserving their biological activity. At the same time, they can protect sensitive tissue from irritating compounds [14]. Despite significant advances in nanoparticles for wound-healing treatment, it is necessary to address their drawbacks. Disadvantages of some nanoparticles include cell damage/toxicity or death, and a negative effect on the wound-healing process using carbon-based nanomaterials [15]; cytotoxicity to mammalian cells [16] at concentrations of 25 μg mL^−1^ or higher using AgNPs [17]; keratinocyte cytotoxicity and exacerbation of allergic skin conditions and skin inflammation [18] using multi-walled carbon nanotube and titanium dioxide nanoparticles (TiO_2_NPs) [19]; skin and ocular irritation using fullerene C_60_ nanoparticles [20]; skin inflammation and psoriasis using ultrafine carbon-based particles [21] and zinc oxide nanoparticles (ZnONPs) [22]; hemolysis in the wound-healing process using AgNPs and ZnONPs [23]. The poly(lactic-co-glycolic) acid polymer (PLGA), on the other hand, is a suitable polymer for producing nanoparticles due to its biocompatibility and biodegradability, approved by the United States Food and Drug Administration (FDA) and the European Medicines Agency (EMA) [24].

The genus *Casimiroa* (*Rutaceae*) includes La (CE) and *Casimiroa pubescens Ramirez*, which are the most relevant species for their medicinal uses. These plants grow in Central America and Mexico. Some studies have been reported investigating the CE therapeutic effects (Table 1). Phenolic compounds [5], fatty acids [6], and sesquiterpene hydrocarbons [8] have been found in CE extracts and its essential oil using different solvents, showing a broad therapeutic potential. Other components found in CE include sugar, proteins, ascorbic acid, phenols, carotenoids, polyunsaturated fatty acids, and minerals (Fe, Cu, Zn, Ca, and K) [25]. Among these extracts, the CE leaf extract in ethanol (a class III solvent, approved for pharmaceutical developments) has been minimally explored, with anticoagulant, neuroprotective, antiapoptotic, and anti-amnesic effects reported [8], opening the window to research. The encapsulation of plant extracts using PLGA for the treatment of wound healing has been underdeveloped [26]. *Phlomis crinita* hydroethanolic extract was encapsulated into PLGA nanoparticles, exhibiting no irritative and no damaging effects on skin integrity. The incorporation of polyethylene glycol (PEG) into the nanoparticles revealed better cell migration and proliferation compared to the free extract, demonstrating the potential for wound-healing treatment [27]. The effectiveness of PLGA nanoparticle ointment containing an ethanol extract of jengkol fruit peel to accelerate the diabetic wound-healing process has also been reported [28]. Another study evaluated the application of ethanolic extract of propolis-loaded nanoparticles on wound healing in diabetic rats, shortening the inflammatory phase and accelerating the cellular proliferation. The improvement through angiogenesis stimulation, fibroblast proliferation, and granulation tissue formation was observed in the early days of the healing phases [29]. Although to a lesser extent, the polymer solution (PLGA) also showed results for wound healing compared to the control group [29]. In these studies, nanoparticles loaded with extracts demonstrated improved solubility, stability, and targeting of the active compounds, enhancing their antimicrobial properties and promoting wound healing in wound environments [2]. The development of biodegradable nanoparticles loaded with CE extract with therapeutic properties for wound healing, among others, is a promising approach that has not yet been reported.

In the present work, CE extract was analyzed by direct injection ion cyclotron Fourier transform mass spectrometry (FIA-ESI-FTICR-MS) untargeted analyses to investigate the extract metabolites. Subsequently, the CE extract was entrapped into biodegradable nanoparticles (CE-NP) and characterized by pH, size, charge, occlusive effect, encapsulation efficiency, morphology, thermal/molecular interactions, dispersion stability, and antioxidant activity. For the first time, stable and spherical CE-NPs with suitable characteristics were obtained, as a preliminary proposal for wound healing.

## 2. Materials and Methods

### 2.1. Materials

Poly (DL-lactide-co-glycolide) 50:50 (viscosity range: 0.15–0.25 dL/g, Mw 24,000–38,000, ACS reagent grade) acid terminated (PLGA); polyvinyl alcohol 4-88 (PVA, EMPROVE^®^, ACS reagent grade), PBS tablets 200 mL of biotechnology grade were purchased from Sigma-Aldrich–Merck KGaA (Burlington, MA, USA) and they were used as the polymer, stabilizer, and as pH regulator, respectively. DPPH (2, 2-diphenyl 1-picryl hydrazyl, 99%) and Trolox (6-hydroxy-2,5,7,8-tetramethylchroman-2-carboxylic acid, 97%) reagents of biotechnology grade were purchased from Sigma-Aldrich Chemical Co. (St Louis, MO, USA). Ethanol, acetone (AC), methyl ethyl ketone (MEK), ethyl acetate (EtAc), hexane, and sodium hydroxide of ACS reagent grade were purchased from Química Suastes, S.A. de C.V., Reactivos Química Meyer (Ciudad de México, CDMX, Mexico). Hydrochloric acid of ACS reagent grade was obtained from J.T. Baker, Phillipsburg, NJ, USA. Ethanol, water, and methanol used for the untargeted phytochemical profiling by FIA-ESI-FTICR-MS were HPLC-grade. Water was obtained from a Millipore System (Darmstadt, HE, Germany).

### 2.2. Plant Material and Extraction Procedure

**Figure 1:**
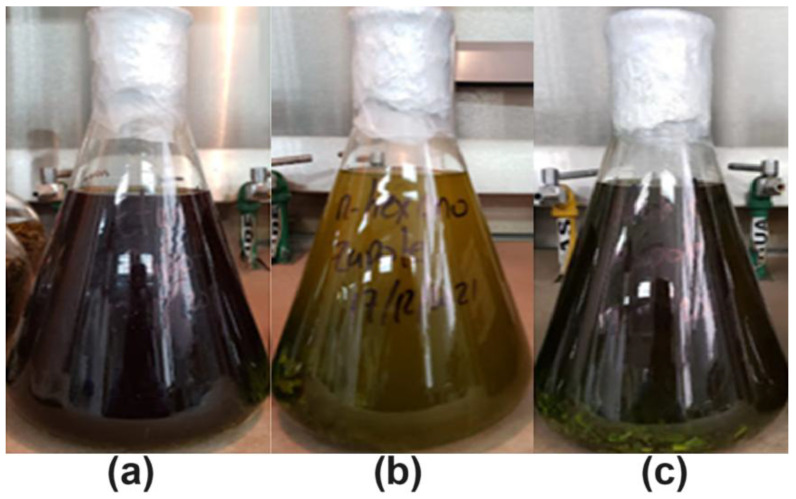
Dried leaves embedded in (**a**) ethanol, (**b**) n-hexane, and (**c**) ethyl acetate after 30 days of storage.

The sapote tree is generally evergreen, retaining its foliage and being available year-round, unlike its fruits or seeds. This abundance is ideal for developing and studying the ethanolic extract of CE leaves, which has been minimally explored. *Casimiroa edulis* La Llave (name given by the International Plant Name Index, IPNI) was obtained from Melchor Ocampo, State of Mexico, Mexico. The taxonomic identity was kindly verified at the Herbarium, Department of Biological Sciences, Faculty of Superior Studies Cuautitlán, National Autonomous University of Mexico (FES-Cuautitlán, UNAM). The leaves were collected in December, from the middle to upper parts of the tree, ensuring that they were free of pests and environmental damage. They were washed with water and dried at room temperature for 7 days and subsequently at 30 °C for 24 h, yielding 48 g of dried leaves. The leaves were ground and extracted with 1 L of 97% ethanol, ethyl acetate, and n-hexane, respectively (Figure 1). After 30 days of storage protected from light, the extracts were concentrated under reduced pressure at 40 °C and 160 rpm using a rotary evaporator to obtain *Casimiroa edulis* yields of 1.151, 1.490, and 3.785 g from n-hexane, ethyl acetate, and ethanol, respectively. These extracts were stored in airtight amber glass containers until further use.

### 2.3. Experimental Methods

#### 2.3.1. Untargeted Phytochemical Profiling by FIA-ESI-FTICR-MS

With the purpose of knowing the number of compounds and their percentage in the CE ethanolic extract, a mass spectrometry study was performed. This untargeted profiling of constituents in the CE extract was carried out by direct-infusion electrospray ionization Fourier transform ion cyclotron resonance mass spectrometry (DI-ESI-FT-ICR-MS) on a Bruker Solarix XR 7 T instrument (Bruker Daltonics, Bremen, Germany). Samples were prepared by dissolving 10 mg of dry extract in 24 mL of water/ethanol (80:20, *v*/*v*) and filtering through a 0.22 µm PES membrane. Infusion was performed with a 250 µL Hamilton syringe at 120 µL h^−1^. Spectra were acquired in both positive and negative ESI modes under the following conditions: capillary 4500 V (2499 nA), end-plate offset −500 V (62.2 nA), 2M acquisition size, *m*/*z* 43–3000, ion-accumulation time 0.31 s, and an average of 100 scans. Nitrogen was used as nebulizer/drying gas (2 bar; 4 L min^−1^ at 180 °C). External calibration employed sodium trifluoroacetate. Data were processed with Compass DataAnalysis 6.0 (Bruker Daltonics), and putative metabolite annotation was performed in MetaboScape 2022b (v9.0.1).

#### 2.3.2. Nanoparticle Preparations

**Figure 2:**
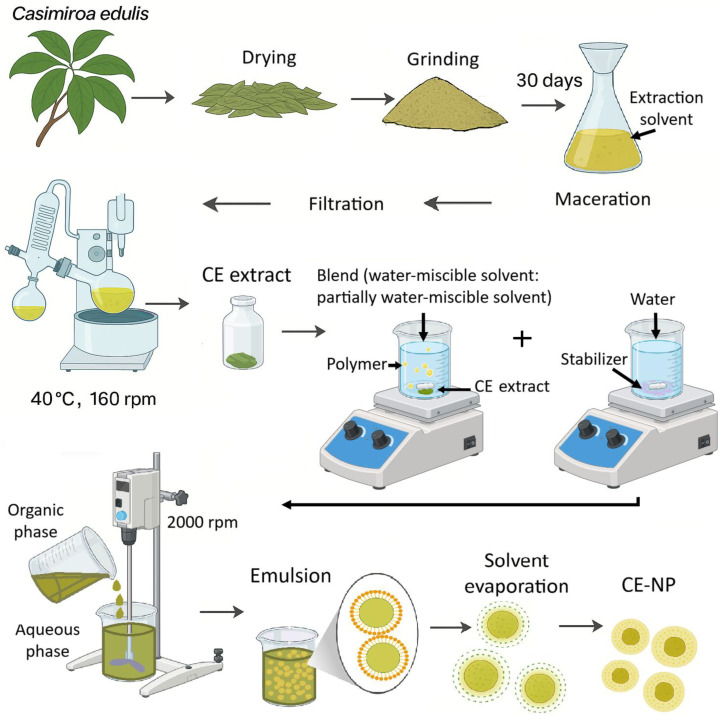
A representative procedure for CE extraction and nanoparticle formation using the rapid emulsion-diffusion method.

To obtain a CE-NP formulation with a size in the nanoscale range and an adequate polydispersity index, previous studies were carried out with different solvent blends and varying amounts of CE. Nanoparticles were prepared by the rapid emulsion-diffusion method [33]. Briefly, 200 mg of PLGA (50:50) and varying amounts (5–100 mg) of CE extracted from ethanol, ethyl acetate, and n-hexane were first dissolved in different solvent blends consisting of a water-miscible solvent and a partially water-miscible solvent in varying ratios. A reaction between the extract and the polymer was observed after nanoparticle preparation, and this problem was mitigated by decreasing the amount of extract and increasing the polarity of the solvent blend. Using a blend of acetone:methyl ethyl ketone (75:25) and 10 mg of CE, a unimodal particle size distribution was obtained in the final dispersion, with improved batch stability only when using CE extracted from ethanol. This organic phase was added dropwise (4 mL/min for 5 min) into an aqueous solution containing PVA at 2% (*w*/*v*), used as a stabilizer, under constant stirring at 2000 rpm with a 2-folding-bladed propeller mechanical stirrer (Science MED (Calgary, AB, Canada), OS20Pro). The emulsification of both phases was followed for 10 min. Finally, the organic solvent was removed by laminar flow for 24 h to reduce the batch instability, which increased when using vacuum steam distillation (Supplementary Figure S1). Batches without the extract were prepared under the same conditions to obtain a placebo (NP-Placebo). A representative procedure for CE extraction and nanoparticle formation is given in Figure 2.

#### 2.3.3. Particle Size Distribution, Particle Charge, and pH Analysis of CE-NP

The dynamic light scattering technique was used to determine the particle size distribution and polydispersity index of nanoparticle dispersions using a Zetasizer (Malvern Systems, Westborough, MA, USA). Measurements were conducted at 25 °C using water viscosity (n = 3). The zeta potential of all batches was determined at 25 °C using the viscosity and dielectric constant of water. Dispersions were diluted with distilled and deionized water as dispersion medium for the particle size and Z potential analysis, respectively, to ensure that the particle count rate was within the instrument’s sensitivity range (>100 and <500), and using an attenuator ≥8 for all measurements (n = 3). pH was determined directly in all suspensions with constant and moderate stirring using a Hanna Instruments pH-meter (Woo0nsocket, RI, USA, n = 3).

#### 2.3.4. In Vitro Antioxidant Activity

The antioxidant activity of CE has been previously reported in hexane and methanol extracts of the fruit’s peel and seeds, but there are no reports of an ethanolic extract of CE leaves. To determine the potential antioxidant activity of the ethanolic extract of CE leaves, and its encapsulation in CE-NP, radical scavenging activity was measured spectrophotometrically against 1, 1-diphenyl-2-picrylhydrazyl (DPPH) following modified methods [34,35]. A DPPH solution in ethanol (200 µM/0.078864 g/L) was used in the reaction mixture. The CE extract was dissolved in ethanol using sonication for 20 min, obtaining a final concentration of 40 µg/mL. The CE-NP dispersion was diluted with water to obtain the same concentration as the CE solution. 450 µL of either the CE solution or the CE-NP dispersion was mixed with 1050 µL of the DPPH solution and incubated at room temperature for 30 min in the dark. After incubation, the absorbance was measured at 517 nm (n = 3). Blank samples were prepared with the same amount of ethanol and DPPH solution. Antioxidant activity is represented as the percentage decrease in absorbance. The percentage of radical scavenging activity of the samples was calculated using the following Equation (1):

(1) $$Radical\;Scavenging\;Activity\;\left(\%\right)=\frac{\left[\left(A_{Blank}-A_{Sample}\right)\right]}{A_{Blank}}\times 100$$

Results were obtained from a Trolox standard curve (y=−28.669x+1.6249, r^2^ = 0.9919) for the DPPH test.

#### 2.3.5. Dispersion Stability Analysis

The physical stability of the CE-NP dispersion was determined using a Turbiscan Classic MA 2000 equipment (Toulouse, France). Pulses of infrared light (880 nm) were applied to 5 mL of a diluted sample (CE-NP dispersion: water, 2:3), placed in a glass tube, and measurements were taken every 16 min for 48 h at room temperature. The average light backscatter difference (ABS%) was plotted and analyzed, along with its variation over time. Profiles of three replicates were analyzed in this study.

#### 2.3.6. FTIR-ATR Analysis

FTIR-ATR analysis was used to determine the molecular interactions of dry CE, CE-NP, and NP-Placebo, as well as for the pure compounds used in the formulations and their physical mixtures, using a Thermo-Nicolet™ iS50 FTIR Spectrometer (Thermo-Fisher Scientific, Waltham, MA, USA). The FTIR was equipped with an attenuated total reflection accessory. Each sample was placed on the germanium crystal surface and compressed to ensure uniform contact between the sample and the crystal. The samples (n = 3) were analyzed from 4000 to 400 cm^−1^, with 64 scans and a resolution of 4 cm^−1^ at room temperature. Representative IR spectrum of samples was used to analyze the peak positions using the peak selection function of the OMNIC 9.12 Hotfix 5 software (Thermo-Fisher Scientific, Waltham, MA, USA).

#### 2.3.7. Encapsulation Efficiency and Drug Loading

Encapsulation efficiency directly impacts the therapeutic effectiveness, safety, and product quality. The percentage of CE encapsulated in the nanoparticle was calculated indirectly as the difference between the total amount of drug added during the nanoparticle preparation process and the amount of CE obtained in the supernatant. The CE-NP dispersion was diluted with distilled water (50:50) and centrifuged at 65,000 rpm for 3 h (centrifuge Ohaus, Frontier 5707, Mexico City, Mexico). An aliquot of the supernatant was filtered through a 0.22 μm PES membrane and read at 264 nm. CE quantification was performed using a UV–Vis spectrophotometer (Eppendorf-BioSpectrometer, Hamburg, Germany, n = 3). CE entrapment was expressed as the ratio of the difference between the total initial amount of CE used in the batch preparation and the amount of free CE present in the supernatant, relative to the total initial amount of CE. It was represented as a percentage, according to the following expression (2):

(2) $$\%\;Encapsulation\;efficiency=\;\frac{Total\;initial\;amount\;of\;CE-Free\;amount\;of\;CE}{Total\;initial\;amount\;of\;CE}\;\times 100$$

The degree of CE loading of the nanoparticles was also determined, which is defined as the ratio between the mass of the encapsulated CE and the CE-loaded nanoparticles. It could be represented by the following expression (3):

(3) $$\%\;Drug\;loading=\frac{Amount\;of\;entrapped\;CE\;in\;CE-NP}{Total\;weight\;of\;CE-NP}\times 100$$

#### 2.3.8. In Vitro Occlusion Test

The occlusive effect is an important characteristic of a healing dressing. An in vitro occlusion test was performed with CE-NP, NP-Placebo, and pure CE using a modified method [36]. Beakers containing 30 g of water were covered with filter paper. A total of 500 μL of NP formulations and a pure extract solution (with the same CE-NP concentration) were slowly spread onto the surface of a filter paper and weighed after drying. A beaker covered with filter paper spread with water was used as a reference. The dripping was carried out uniformly over the entire surface of the paper, allowing it to dry while preventing it from falling into the water in the beaker. The beakers were stored at 32 °C to mimic the temperature of the skin surface, and their weights were registered at 12, 24, and 48 h (n = 3). Water evaporation through the filter papers was calculated in terms of water loss. The occlusion factor (*OF*) was calculated using the following Equation (4):

(4) $$OF=\frac{R-S}{R}\times 100$$

where *R* = reference water loss and *S* = sample water loss. An *OF* value of 0 indicates the absence of occlusive effect, while an *OF* value of 100 indicates maximum occlusion.

#### 2.3.9. Morphology Studies

CE-NP were examined morphologically to know their structure, size, and form using a JEM2010 Transmission Electron Microscope (Jeol Ltd., Tokyo, Japan). Samples were diluted with distilled water (1:10) and were placed on copper grids and dried under laminar flow. Images were obtained utilizing a voltage of 200,000 V and a resolution of 6000–40,000 X. Three samples were observed by scanning the entire grid, particularly the fields near the corners, and at least 3 images were obtained of each sample. Representative images were selected in this study.

### 2.4. Statistical Analysis

The results were statistically evaluated using analysis of variance (ANOVA), one way to compare two or multiple groups. Post hoc comparisons of the means of each group were performed by applying the Bonferroni test for one or more factors. Differences were considered significant if the *p*-value < 0.01. Software STATGRAPHICS^®^ Centurion XVI was used for this purpose.

## 3. Results and Discussion

In general terms, interesting compounds such as tetramethylscutellarein, rutin, S-usnate and a eugenol derivative with a wide range of reported therapeutic activities (antioxidant, anti-inflammatory, antibacterial, hemostatic and wound-healing properties, but also modulator of bacterial drug resistance) were found in the CE extract obtained from ethanol, and good physicochemical properties of the CE-NP were obtained with a mainly antioxidant effect which could help in wound-healing treatment.

CE extraction yields of 1.151, 1.490, and 3.785 g from n-hexane, ethyl acetate, and ethanol, respectively, were obtained, but only the CE extracted from ethanol was suitable to prepare nanoparticles with adequate stability. Here, CE extracted with ethanol, and CE-NP were further characterized.

### 3.1. Untargeted Phytochemical Profiling by FIA-ESI-FTICR-MS

An exploratory untargeted analysis of the CE extract was performed to profile phytochemical classes and their relative abundances, to contextualize them with previously reported therapeutic properties. Metabolites were profiled using the ultra-high resolution of FT-ICR-MS. After the CE extract analysis, 40 ions in positive and 34 ions in negative electrospray ionization modes (ESI+ and ESI−, respectively) were matched to database formulas by accurate mass with an error tolerance of ≤10 mDa (Table 2; Table 3) and <100 mDa (Supplementary Tables S1 and S2).

**Figure 3:**
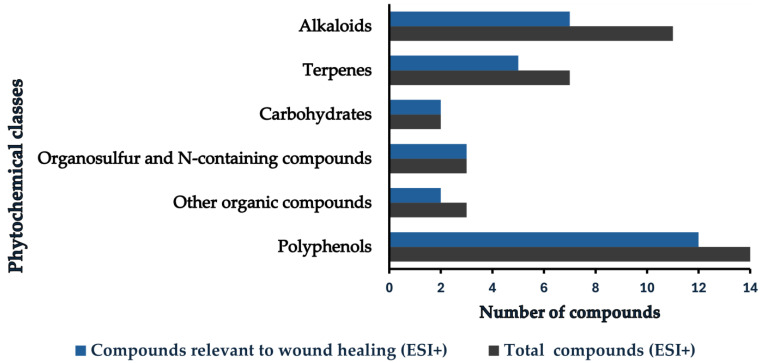
Distribution of phytochemical classes identified in positive electrospray ionization mode (ESI+) associated with wound-healing activity. The compounds relevant to wound healing were selected because they exhibited anti-inflammatory, antioxidant, antimicrobial (antibacterial), antibiotic, and antifungal activities in previous studies, and other effects that aid the wound-healing process.

**Figure 4:**
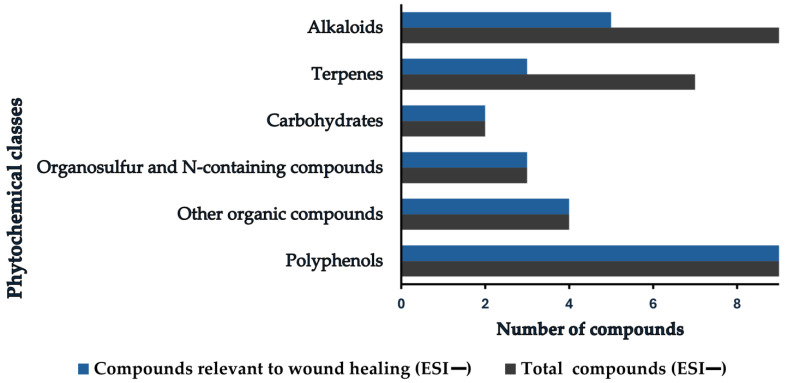
Distribution of phytochemical classes identified in negative electrospray ionization mode (ESI−) associated with wound-healing activity. The compounds relevant to wound healing were selected because they exhibited anti-inflammatory, antioxidant, antimicrobial (antibacterial), antibiotic, and antifungal activities in previous studies, and other effects that aid the wound-healing process.

Within the ≤10 mDa tolerance, phytochemicals in the CE extract were grouped into alkaloids, carbohydrates, polyphenols, terpenes, organosulfur compounds, N-containing compounds (such as glucosinolates, isothiocyanates, amino acids, proteins, nucleosides, and nucleotides), and a miscellaneous set of other organics (including fatty acids, lipids, organic acids, and vitamins). Polyphenols were further organized into alkylresorcinols, bromophenols, flavonoids, lignans, miscellaneous polyphenols, phenolic acids, and tannins. Figure 3 and Figure 4 summarize these constituents, highlighting polyphenols as the predominant class in the CE extract.

Tetramethylscutellarein (4′,5,6,7-tetramethoxyflavone)—a scutellarin derivative—was the most abundant polyphenol (40.34%). Consistent with this result, previous studies have also identified 5,6,2′,3′,5′,6′-hexamethoxyflavone and 5,6,2′-trimethoxyflavone in methanol–chloroform extracts of CE leaves [7]. Several therapeutic activities have been reported for tetramethylscutellarein: in vitro, it accelerates blood coagulation via the intrinsic pathway, probably involving the reaction of factors XII, XI, IX, or VIII [37]; tetramethylscutellarein (tetramethyl-O-scutellarin) isolated from immature Shiranuhi citrus peels exhibits anti-inflammatory effects on skin by suppressing the release of pro-inflammatory cytokines like IL-6, IL-1β, TNF-α, NO, and PGE2 and inhibiting NF-κB and MAPK signaling pathways [38]; and additional work has shown anti-inflammatory action via inhibition of p65 nuclear translocation from the cytoplasm [39]. These activities suggest potential for wound healing and for treating inflammation-related skin conditions such as psoriasis, dermatitis, and acne. Antibacterial effects have also been reported against four diarrheagenic pathogens (*Klebsiella oxytoca*, *Salmonella enterica*, *Shigella sonnei*, and *Vibrio cholerae*) [40]. Although no direct activity was observed against *Staphylococcus aureus* SA-1199B, a NorA efflux pump–overexpressing strain, tetramethylscutellarein modulated bacterial drug resistance by reducing the minimal inhibitory concentration of norfloxacin 16-fold [41]. A wound-healing-associated effect was also reported when several flavonoids, including tetramethylscutellarein, were tested in a dorsal rat model [42].

The second most abundant compound (13.53%) was rutin, a flavonoid that has shown efficacy against *Escherichia coli*, *Pseudomonas aeruginosa*, and *Staphylococcus aureus* in previous studies [43,44]. In combination with gentamicin, additive effects on epithelial regeneration were found, with superior control of biofilm and enhanced wound-healing activity in diabetic infections [43]. Rutin has also been promoted for wound repair by attenuating oxidative stress (restoring GSH, CAT, and SOD levels and decreasing MDA production) mediated by Nrf2 activation after topical administration in a rutin-nanoparticle formulation [45]. A multifunctional chitosan hydrogel modified with 4-carboxyphenylboronic acid and loaded with rutin exhibited a similar potent intracellular free-radical-scavenging activity (53–70%), an increased collagen deposition, and a promotion of angiogenesis [44]. Rutin has also been reported as anti-inflammatory by decreasing CRP, IL-1β, IL-6, and TNF-α levels while increasing anti-inflammatory markers (Arg1 and CD206) in RAW 264.7 macrophages [44]. Rutin, together with quercetin and human umbilical cord-derived mesenchymal stem cells, showed a synergistic effect on wound healing by reducing inflammation, mitigating oxidative stress, and enhancing neovascularization in cold burn wounds (wounds with hypertrophic scars, contracture, and necrosis) [46].

Eugenyl-GXG (eugenol 2-O-β-D-glucopyranosyl-(1→2)-(O-β-D-xylopyranosyl-(1→6))-O-β-D-glucopyranoside), a polyphenolic eugenol derivative, was detected in the CE extract at 3.05%. Although its specific biological properties have not yet been characterized, they can reasonably be related to those of eugenol [47] and acetyleugenol [48] (antibacterial, antiviral, antifungal, anticancer, anti-inflammatory, and antioxidant [49]). Another polyphenol, rotenone (found in the CE extract at 2.69%), is considered safe when properly used. Rotenone exhibits insecticidal, acaricidal, and other pesticidal activities [50], as does 2-tridecanone, another insecticidal compound found at low abundance (0.38%). Caffeic acid 4-O-glucoside or linocaffeine (found at 2.44%), previously showed significant antioxidant capacity, inhibition of advanced glycation end-products (AGEs-implicated in aging, diabetes, uremia, cataracts, atherosclerosis, and Alzheimer’s disease), and inhibitory activity toward acetylcholinesterase and angiotensin-converting enzymes, with potential to cause cardiovascular and neurodegenerative disorders [51]. Cinnamoyl-glucose (found at 1.70%) has been reported with a antibacterial associated effect against *E. coli* and antioxidant effects [52], as well as to suppress fibroblast apoptosis induced by carboxymethyl-lysine–collagen at 10 μg/mL, being effective against skin AGE formation and fibroblast damage [53]. The flavonoid Vicenin-2 (found at 1.2% in the CE extract) has been reported as an antioxidant, renoprotector [54], antiseptic [55], anti-inflammatory [56], anticancer, anti-proliferative, anti-angiogenic, pro-apoptotic [57], trypanocidal [58], antispasmodic [59,60], antinociceptive [58], anti-diabetic, and hepatoprotective [61]. Vicenin-2 has also shown efficacy in anti-glycation [62], diabetic wound healing [63], and in preventing skin photoaging [64]. Prodelphinidin B3 and kaempferol 3-O-(6-acetyl-galactoside)-7-O-rhamnoside were both present at <1% in the CE extract. Prodelphinidin B3, reported with anti-inflammatory [65] and antioxidant activities [66], showed an associated effect to inhibit prostate cancer cell growth through cell-cycle arrest and caspase-3 activation [67]. On the other hand, the kaempferol derivative has been studied, showing antioxidant properties [68], antiproliferative activity against HepG2, CT26, and B16F1 (human hepatoma, mouse colon cancer, and mouse melanoma, respectively), and an inhibitor of AKT phosphorylation and cleaved caspase-9, caspase-7, caspase-3, and PARP in HepG2 cells, showing anti-inflammatory action [68].

In general, polyphenols are recognized for their robust anti-glycation capacity [53]. Flavonoids are antioxidants that provide immune protection against environmental and endogenous toxins. Numerous preclinical studies (predominantly in murine models) report anticancer, antibacterial, antifungal, anti-diabetic, antimalarial, neuroprotective, cardioprotective, and anti-inflammatory activities, and a reduced risk of diabetes mellitus, cancer, stroke, and myocardial infarction [69]. Lignans—a subclass of polyphenols—are known to exert antioxidant and anti-inflammatory effects and act via estrogen receptor-dependent pathways [70]. Phenolic acids, another subclass of polyphenols, possess resonance-stabilized phenolic structures that support hydrogen-atom donation and radical scavenging. Electron transfer and singlet-oxygen suppression also contribute to the antioxidant activity of phenolic acids, alongside reported antimicrobial, anticancer, anti-inflammatory, and antimutagenic effects [71].

Several other organic constituents were identified in the CE extract. (S)-Usnate (C18H14O7)—the deprotonated derivative (salt) of usnic acid (C18H16O7)—was the third most abundant compound (4.03%). Although data on the isolated (S)-enantiomer are lacking, it may be hypothesized that it enhances the therapeutic profile (antibacterial, immunostimulatory, antiviral, antifungal, anti-inflammatory, antitumor, antimicrobial, and antiparasitic) of usnic acid/usnate salts, while potentially reducing toxicity [72,73,74]. Protoporphyrin IX (present at 2.28%) is involved in the porphyrin scaffolding of heme b, oxygen binding (hemoglobin, myoglobin), electron transfer (cytochrome c), and catalysis (cytochrome P450, catalases, peroxidases) [75]. Coniferin (found at 1.93%) promotes apoptosis in fibroblast-like synoviocytes via the PTGS2 of rheumatoid arthritis with anti-inflammatory, antioxidative, and mitochondrial transmembrane-potential-disrupting effects [76].

Plant carbohydrates—classified as mono-, oligo-, and polysaccharides according to their degree of polymerization—have been reported to exhibit antioxidant, antitumor, immunostimulatory, antibacterial, anticoagulant, and hypoglycemic activities [77]. Lactose (3.60%) and primeverose (1.94%, no therapeutic studies have been reported) were detected in the CE. Lactose has demonstrated anti-aging effects by decreasing elastase synthesis/release and inhibiting fibronectin secretion by fibroblasts in in vitro models [78]. Topical administration of retinol (0.1%), lactose (5%), and glycolic acid (4%) improved the appearance of photodamaged skin [79].

Among the compounds containing N in CE, betaxanthins were observed to show antimicrobial activity in a previous work [80]. Specifically, 3-methoxy-tyramine-betaxanthin, threonine-betaxanthin, and methionine-betaxanthin were active against Gram-positive bacteria (*Staphylococcus aureus*, *Staphylococcus epidermidis, Micrococcus luteus*, *Enterococcus faecalis,* and *Bacillus cereus*/*subtilis*); Threonine- and 3-methoxy-tyramine-betaxanthins were also active against some Gram-negative species (*Salmonella Typhimurium*, *Proteus mirabilis*, *Bordetella bronchiseptica*, *Escherichia coli*, and *Pseudomonas* aeruginosa); whereas methionine-betaxanthin only inhibited *P. mirabilis*, *B. bronchiseptica*, and *Klebsiella. pneumoniae* [80]. Antifungal activity was also reported against *Candida parapsilosis, Candida albicans, Candida glabrata, Candida krusei, Candida lusitaniae* and *Candida tropicalis* for 3-methoxy-tyramine-betaxanthin, threonine-betaxanthin, and methionine-betaxanthin [80]. Sinapoyltyramine (0.60% in CE extract), another N-containing constituent, has also shown moderate antimicrobial and antifungal activities [81].

Alkaloids—secondary metabolites encompassing pyridine, tropane, isoquinoline, phenanthrene, phenylethylamine, indole, purine, imidazole, and terpenoid subclasses—are widely recognized for their anti-inflammatory, antitumor, antibacterial, antifungal, and antiviral properties [82]. The most abundant alkaloid in CE, magnoflorine (1.24%), has been reported to exhibit antidiabetic, antioxidant, anti-inflammatory, immunomodulatory, anticancer, relaxant, anti-osteoporotic, antibacterial, antifungal, and antidepressant effects [83,84,85,86].

Lipids were also detected in the CE. Fatty acids can be classified according to chain length (short-, medium-, long-chain) with their corresponding bioactivities: short-chain species have been reported to exert immunoregulatory effects; medium-chain species generate ketogenic metabolites; and long-chain species produce oxidized metabolites with applications in metabolic disorders [87]. Regarding polar lipids (phospho-, glyco-, and sphingolipids), they have previously shown anti-inflammatory effects at all stages of atherosclerosis and cardioprotective properties by modulating the platelet-activating factor axis. Bioactive lipids can also inhibit the pro-inflammatory activities of platelet-activating factor, modulating its metabolism toward homeostasis [88]. Among prenyl-lipids, plastoquinol-9 (0.36% in CE extract) is an effective singlet-oxygen scavenger in vitro and in vivo, and outperformed tocopherols in inhibiting lipid peroxidation [89].

Terpenes—volatile secondary metabolites produced by plants, animals, insects, and other organisms—were present at low abundance in the CE extract. Plants synthesize more than 55,000 terpenes, primarily as constituents of essential oils. Biological activities of terpenes include antifungal, antiviral, antimicrobial, anti-inflammatory, antiparasitic, antihyperglycemic, anticancer, and analgesic effects [90].

Finally, additional organic compounds identified in the CE extract have been previously reported to have pharmacological effects relevant to wound healing. Stilbenoids such as dehydro-glucosyl-piceatannol have exhibited antioxidant, antimicrobial, anti-diabetic, anti-inflammatory, antiproliferative, cardioprotective, and neuroprotective activities [91]. Pheophorbide-α, a chlorophyll-derived photosensitizer with antioxidant, anti-inflammatory, and phototoxic properties, has been widely reported to enhance photodynamic therapy in breast, prostate, lung, oral squamous cell, gastric, osteosarcoma, and cervical cancers [92]. 3-Isobutanoyl-4-(3-methylbutanoyl) sucrose was also detected in the CE extract, and in combination with other metabolites (acyl sucrose esters, withanolides, and flavonoids) from the calyces of *P. peruviana* L. showed immunostimulatory, anti-inflammatory, and antiapoptotic associated activities [93]. Low levels of thiamine, glutathione, and dimethyl trisulfide were present in the CE extract. Glutathione is a key redox buffer with the ability to quench free radicals in response to oxidative stress [94]. Dimethyl trisulfide has demonstrated antifungal activity and biocontrol against *Alternaria alternata* on postharvest *Lycium barbarum* [95].

Of all these phytochemical compounds found in CE, most have effects that help indirectly and directly in wound healing, such as antioxidant, anti-inflammatory, antibacterial, antifungal, hemostatic, healing, moisturizing, and drug-resistant bacterial properties.

### 3.2. CE-NP Characterization

Nanoparticle batches were prepared first using a solvent blend of acetone: ethyl acetate (50:50) as the organic phase, with different amounts (5–100 mg) of CE. However, physical instability and a probable reaction between the extract and the polymer led to the presence of a cloudy yellow precipitate after the nanoparticle preparation in all batches, particularly in batches 1–6 with increased addition of CE, as observed in Table 4. In these batches (1–6), multimodal populations of particles at the micro and nano scale were obtained. The use of a solvent blend of acetone:methyl ethyl ketone (75:25) with different amounts of CE extract improved the physical stability in batches 7–10, and dispersions with a unimodal distribution of particle size at the nanoscale range with adequate polydispersity index were obtained. As the solvent blend polarity increased in the organic phase (OP), the CE solubility decreased. Using CE extracted from hexane and ethyl acetate to produce nanoparticles, a precipitate was observed adhering to the glass container in which the nanoparticles were prepared. Batch 7, on the other hand, showed enhanced stability, entrapping 10 mg of ethanolic CE extract. Formulation stability was also enhanced when the organic phase was gradually removed by laminar flow for 24 h instead of using the vacuum steam distillation. Batch 7 was further characterized and tested to evaluate the antioxidant capacity.

Physical instability of the nanoparticle batches between the extract and the polymer could be explained by the degradation of aliphatic *co*-polyesters (PLGA 50:50), through the hydrolysis of their ester bonds and their interaction with polyphenols found in CE [96]. PLGA degrades due to its amorphous structure and the presence of more hydrophilic glycolide monomers in the chain, in this case, 50% in the polymer used. Degradation can be significantly affected by PLGA characteristics (molecular weight, degree of crystallinity, wettability, surface area, porosity, and monomer ratio), and the presence of drugs, phytodrugs, ceramic particles, and polymers, but also depends on external factors, such as pH, temperature, and mechanical stress [97].

Nanoparticle production involved the energetic mixture of a PVA aqueous solution and the polymer solution at 2000 rpm, increasing the hydrophilicity rapidly. This degradation of PLGA may lead to the generation of acidic by-products, increasing the acidity of the environment, promoting PLGA autocatalysis, and accelerating the rate of degradation. Polyphenols, because of the presence of an aromatic ring and a hydrogen atom of the phenolic hydroxyl group, are weak acids [96]. It could be hypothesized that both hydrophilicity and the acidic character of the CE polyphenols caused a little degradation of PLGA, producing some big particles. This explains the increase in instability in the nanoparticle batches as CE increased. In a previous study, it was shown that acidic drugs such as aspirin accelerated hydrolysis of ester bonds of PLGA through acid catalysis, while hydrophilicity facilitated the water absorption [98]. Reducing mechanical stress by gradually removing the organic solvent, instead of using vacuum steam distillation, also contributed to the stability of the formulation. On the other hand, polyphenols interact with molecules like carbohydrates, lipids, and proteins; the interactions can result in the creation of bigger associations. The binding is mostly hydrophobic, although hydrogen bonds and covalent bonds can be created, too [99]. These associations could probably be formed in nanoparticle production since CE contains a considerable percentage of carbohydrates and lipids. This also could explain the higher instability produced when CE extracts from hexane and ethyl acetate (containing more hydrophobic compounds-e.g., lipids) were used to prepare nanoparticles.

PLGA degradation makes the polymer useful in biomedical applications because of its biocompatibility and biodegradability. The United States Food and Drug Administration (FDA) and the European Medicines Agency (EMA) have approved various PLGA particle formulations as therapeutic delivery vehicles [24].

**Figure 5:**
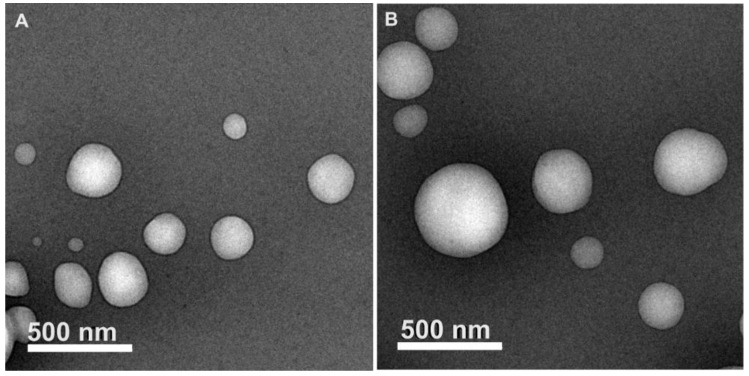
TEM micrographs of CE-NP prepared with PLGA (50:50). (**A**) Spherical and solid nanoparticles with a particle size of ~200 nm. (**B**) Nanoparticles with smaller and larger particle sizes than those obtained by the DLS technique. The bar scale represents 500 nm.

The particle size of the selected batch (7) obtained by dynamic light scattering was 169.30 ± 1.30 nm and was very similar to the particle size obtained by TEM (Figure 5). TEM micrographs could also show that CE-NP are solid and spherical, which is common in nanoparticles obtained by emulsion-diffusion methods using PLGA [100]. An adequate entrapping of CE extract was obtained, 57.00 ± 0.74% with a drug loading content of 1.62 ± 0.02% for batch 7. The entrapping percentage is good and suitable to protect from light or oxygen at least half of the amount of the CE extract (contains phytodrugs) entrapped into the nanoparticles, preventing oxidation and the subsequent inactivity of bio-actives.

### 3.3. In Vitro CE Antioxidant Activity

The antioxidant activity of CE was evaluated by determining the percentage radical scavenging activity of samples containing CE. Results showed that pure CE at a low concentration (40 µg/mL) possessed a good antioxidant activity (29.04 ± 5.16%, 17.27 ± 2.86 μg/mL) when DPPH radical was used. CE-NP diluted dispersion at the same concentration (40 µg/mL) showed a similar antioxidant activity (28.06 ± 2.30%, 16.73 ± 1.28 μg/mL). These results agree with those previously determined from *Casimiroa edulis* extracted with methanol of peel fruit [6]. The CE antioxidant activity in this study was ~2-fold higher than that obtained from a CE methanolic extract of seeds [5], which is suitable for wound-healing therapies. The steps of wound healing include hemostasis, inflammation, proliferation, and matrix remodeling. Reactive oxygen species (ROS) play a fundamental role in regulating antimicrobial defense, platelet activation, and angiogenesis. However, excessive ROS levels can induce oxidative stress, disrupt the healing cascade, and contribute to chronic wounds, inflammation, and impaired tissue repair. Diabetes, obesity, smoking, and aging further exacerbate oxidative stress and consequently the wound-healing process. Antioxidants have demonstrated therapeutic potential in mitigating oxidative stress, promoting wound closure, and enhancing cellular recovery [101].

Another mechanism of antioxidants to promote wound closure is through the inhibition of endopeptidases responsible for the degradation of the extracellular matrix (ECM). Matrix metalloproteinase-1 (MMP-1) is an endopeptidase classified as a collagenase, responsible for the degradation of collagen in the ECM [102]. Wound healing stimulates the expression of endopeptidases, especially MMP-1. Fibroblasts also stimulate the ROS production, which further secretes peroxide ions and superoxide ions. The synthesized ROS generate various cytokines, increase the proteinase production, and alter the fragments of the ECM [103]. Substances acting as MMP-1 inhibitors (exhibiting anti-elastase and anti-collagenase), such as antioxidants, can be potential modulators of the free radical-induced cellular damages [104].

Although our study was carried out under in vitro conditions, providing excellent results, it is suggested to perform an evaluation of intracellular reactive oxygen species (ROS) scavenging and cell-based studies as part of future investigations.

### 3.4. Dispersion Stability Study of CE-NP

**Figure 6:**
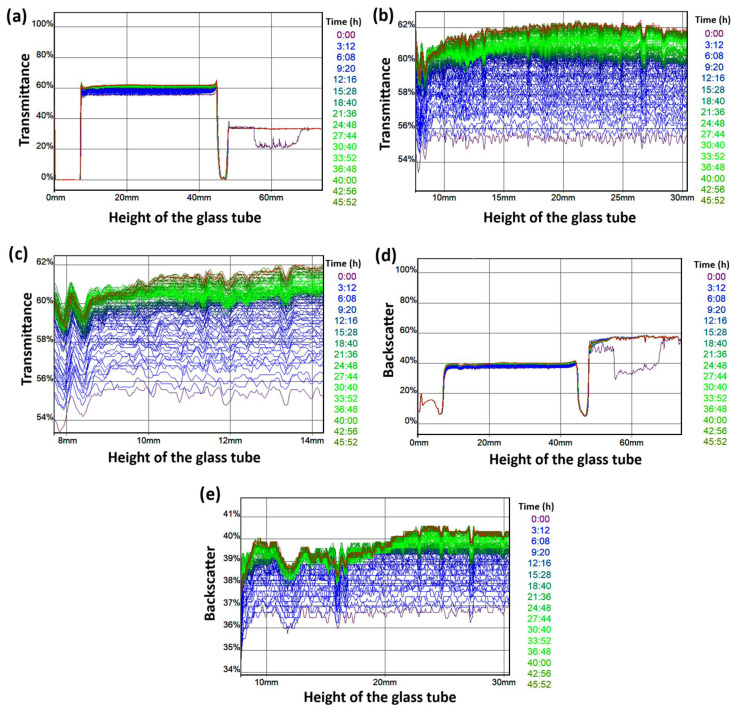
(**a**) Transmittance profiles of 5 mL of a diluted sample of nanoparticles (CE-NP dispersion: water, 2:3; n = 3); (**b**,**c**) amplifications of the transmission profiles showing minimal differences of transmittance. (**d**) Back scattering profiles of a diluted sample of CE-NP dispersion and their (**e**) amplifications.

This study was performed on CE-NP prepared with CE from ethanolic extract to analyze the transmission and backscattering (BS) light profiles. Results show very small differences between the profiles during 48 h of the study, as observed in Figure 6. A similar tendency was observed for all profiles with a light displacement with respect to time, which indicates a good stability of the sample. Profiles amplifications show minimal changes with the same tendency (Figure 6b,c,e). It is important to mention that a diluted sample of CE-NP (CE-NP dispersion: water, 2:3) was used in this study, suitable to be analyzed by the equipment. This sample with decreased viscosity and lower steric effects between the nanoparticles maintained good physical stability, free of coalescence and flocculation after the study. Then, an even better physical stability for the CE-NP dispersion without any dilution could be inferred. This result agrees with the adequate Z potential and excellent PdI obtained for the nanoparticles entrapped with CE (Table 1).

### 3.5. Occlusion Test

**Figure 7:**
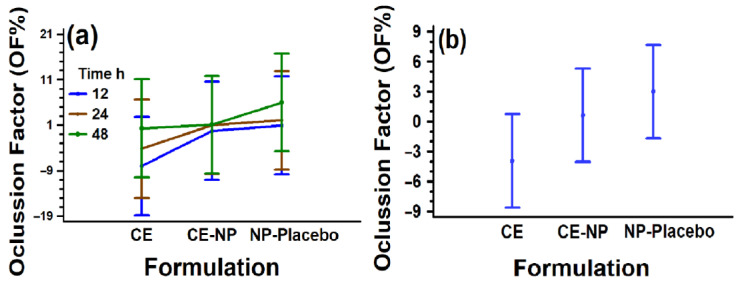
(**a**) Occlusion factor of the pure extract (CE), the nanoparticle dispersion (CE-NP), and the placebo nanoparticle dispersion (NP-Placebo) at 12, 24, and 48 h. (**b**) Occlusion factor average of formulations from data obtained in all timepoints (n = 3). All the means bars correspond to the Bonferroni interval at the 99.0% confidence level (*p* value = 0.0629 for the formulation effect; *p* value = 0.2374 for the time effect.

The occlusion factor (*OF*) is a parameter that indicates the percentage of water retained at a specific time. The results obtained in this study showed no significant differences (*p* < 0.01) for all formulations tested, which could be due, on the one hand, to the high variation as observed in Figure 7. On the other hand, the modification of the method to determine the occlusive effect, in which a paper filter was replaced instead of a metal mesh, could have retained more water in the beakers, reducing the differences. It is important to mention that even when the results did not show significant differences in *OF* means, there is a tendency of nanoparticle formulations (including the placebo batch) to provide a higher occlusion factor compared to the reference (control). CE-NP could offer a suitable occlusion as dressing for 24–48 h for wound-healing treatment. The importance of providing an occlusive effect by occlusive dressings for the regeneration of the skin is crucial due to the increase of up to ~40% of the process of re-epithelialization of partial-thickness wounds [105]. Occlusion shortens the inflammatory healing phase and produces good cosmetic results, contrary to the dry wound, which tends to heal slowly with poor cosmesis [106]. Results of the occlusive properties determined in vitro/ex vivo may differ from the in vivo method, but they offer a good indication of occlusion, as it was previously reported [36]. It is suggested that these studies could be carried out in vivo to confirm the results.

### 3.6. IR ATR Studies

**Figure 8:**
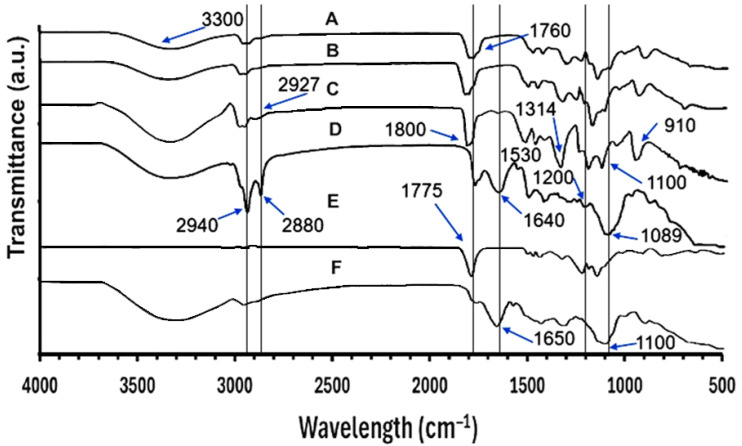
Infrared spectra of the CE-NP patches and their components and physical mixtures: (A) CE-NP; (B) NP-Placebo; (C) PVA; (D) pure CE; (E) PLGA 50:50; (F) physical mixture of PLGA 50:50 + PVA + CE.

FTIR studies were conducted to investigate possible interaction between the components before and after undergoing processing during CE encapsulation. Figure 8 shows the infrared spectra, which display typical bands of the polymers [107], and the molecular interaction primarily between PLGA 50:50 and the CE extract. Analyzing the pure PLGA 50:50 spectrum (E), it displays an intense and narrow band at ~1775 cm^−1^, corresponding to C=O stretching of ester groups. CE (D), on the other hand, presents bands at 2940 and 2880 cm^−1^ attributed to C–H stretches of –C–H [108] of methyl and methylene groups, probably from the most abundant compound in the CE, tetramethylscutellarein (a flavonoid with four methyl ether groups). Signals at 1640 and 1089 cm^−1^ were associated with aromatic C=C stretching and with C–O stretching of ester and ether bonds, respectively [107]. Regarding PVA peaks, they are similar with those reported previously [109] observed at 3300, 2927, 1800, 1425, 1314, 1100 and 910 cm^−1^, corresponding to O–H stretching vibration of the hydroxy group, CH_2_ asymmetric stretching vibration, C=O carbonyl stretch, C–H bending vibration of CH_2_, C–H deformation vibration, C–O stretching of acetyl groups and C–C stretching vibration, respectively [109]. These signals of pure compounds in the physical mixture (F) appear in an additive manner, indicating the absence of relevant chemical interactions. In the opposite sense, a broadening and slight shift in the carbonyl band (C=O, ~1760 cm^−1^) compared to PLGA (E) is observed in the FT-IR spectrum of CE-NP (A), accompanied by an enlargement of the O–H band (3400–3200 cm^−1^). This suggests the formation of hydrogen-bonding interactions between the carbonyls of PLGA and the hydroxyl groups of CE phenols. This behavior is also found in the 1200–1000 cm^−1^ region corresponding to C–O–C vibrations (esters and ethers), where variations in intensity and better-defined bands appear, confirming the existence of additional interactions between the polymer and the bioactive compounds. Similar interactions were found for PLGA and polyphenols in another study [96]. Bands at 1640 cm^−1^, 1530, and 1089 cm^−1^, arising from CE polyphenols, did not appear or were attenuated in CE-NP spectra, indicating that surfaces of the nanoparticles were free of CE and their subsequent encapsulation. This agrees with the results of another study entrapping caffeic acid phenethyl ester into PLGA nanoparticles [110]. The modifications observed in the C=O, O–H, and C–O–C bands confirm that, unlike the physical mixture, the CE-loaded nanoparticles exhibit specific molecular interactions that favor the encapsulation and stabilization of CE bioactives within the PLGA polymeric matrix.

Biodegradable nanoparticles encapsulated with 10 mg of CE were developed and characterized, exhibiting favorable physicochemical properties, as a preliminary approach to wound healing. Advances in nanotechnology using natural products have demonstrated improved performance in wound treatment, enhancing their bactericidal, antioxidant, antimicrobial, anti-inflammatory, and regenerative properties [111]. Similarly, it could be hypothesized that compounds present in CE, such as tetramethylscutellarein, rutin, S-usnate, and a eugenol derivative, could enhance their therapeutic activities (antioxidant, anti-inflammatory, antibacterial, hemostatic, and wound-healing properties, as well as modulate bacterial drug resistance and promote collagen synthesis) when encapsulated in nanoparticles. CE-NP is a preliminary proposal offering a more sustainable therapy for wound healing using a biodegradable polymer and an extract from a class III solvent (ethanol) with a broad therapeutic spectrum, according to reported research. This study confirmed the superior antioxidant activity of CE and CE-NP, which could help in the treatment of wound healing, but this nanotherapy could also be used for other applications.

## 4. Conclusions

The ethanolic extract of *Casimiroa edulis* leaves was characterized by mass spectrometry (FIA-ESI-FTICR-MS) and encapsulated in biodegradable nanoparticles as an alternative to overcome the common problems related to efficacy and toxicity of nanotherapies. Exploratory FI-ESI-FTICR-MS analysis of the *Casimiroa edulis* leaf ethanolic extract showed 40/34 ions in positive/negative electrospray ionization modes, putatively annotated by accurate mass against databases with an error tolerance of ≤10 mDa. The most abundant compounds showed the following order: tetramethylscutellarein > rutin > S-usnate > lactose > a eugenol derivative > rotenone; polyphenols in predominant amount, carbohydrates, depsidones/other phenolics, etc., were also detected. Biodegradable *Casimiroa edulis*-loaded nanoparticles of PLGA (50:50) were produced by the rapid emulsion-diffusion method using an acetone:methyl ethyl ketone mixture (75:25) as the organic phase. The nanoparticles showed a suitable particle size with excellent polydispersity index, charge, occlusive effect, and good dispersion stability, which was confirmed by Z potential, transmittance, and FTIR-ATR studies. The nanoparticles also showed a favorable tendency to provide an occlusive effect compared to the pure extract. The antioxidant activity of the extract/nanoparticles at 40 µg/mL was 17.27 ± 2.86/16.73 ± 1.28 μg/mL, two-fold higher than that previously reported from sapote seeds. For the first time, stable, spherical CE-NP with suitable characteristics were obtained as a preliminary proposal for wound healing, without being limited to other applications. This therapeutic approach could subsequently help millions of patients worldwide, preventing complications such as long-term disability or even death caused by failed nanotherapies. Efficacy, toxicity, and safety studies should be conducted.

## Figures and Tables

**Table 1:** Phytochemical studies previously reported on *Casimiroa edulis* La Llave et Lex (CE).

Part of the Studied Plant	Solvents and Conditions of Extraction	Identified Compounds	Therapeutic Effects	Ref.
Leaf extract	Ethanol with fractions in diethyl ether, chloroform, ethyl acetate, and n-butanol.	Coumarins (umbelliferone, esculetin, imperatorin, and xanthotoxol)	Anticoagulant activity	[8]
Leaf extract	Methanol with subsequent fractions of chloroform–methanol with methanol increases from 0 to 10%	Coumarins and polymethoxyflavones	Adipogenesis activity	[7]
Leaf essential oil	—	Sesquiterpene hydrocarbons	Antimicrobial activity comparable to chloramphenicol and kanamycin	[8]
Seed extract	Methanol for 24 h	Phenolic compounds (herniarin, imperatorin, 8-geranyloxypsoralen, and 5,6,2′,3′,4′-pentamethoxyflavone)	Vasorelaxation effect on arterial tissues; scavenging activity in a concentration-dependent manner; and synergism of vasodilation and antioxidant activities.	[5]
Fruit peel and seed extract	n-Hexane followed by methanol (72 h, 60 °C)	Phenolic compounds and fatty acids	Antioxidant, anti-inflammatory (higher than diclofenac), and antitumor activities.	[6]
Seed extract	Water solid dispersion, at room temperature	—	Anticonvulsant, hypnotic, antihypertensive, diuretic, and sedative activities	[30]
Leaf extract	Methanol was previously defatted with petroleum ether at room temperature, and then fractionated with methylene chloride and ethyl acetate.	Flavonoids and other polyphenolic compounds	Mitigator of the toxicity induced via AgNPs	[10]
Leaf extract	Methanol	4,6-Dimethoxy−3-(4-methoxybenzylidene) benzo(B)furan−2-one, glycerol 1-palmitate, n-hexadecanoic acid, 2-pentanone, 4-hydroxy−4-methyl, and cis-vaccenic acid.	Reduces cholesterol levels, enhances the activity of testicular enzymes, and restores testicle structure, improving sperm quality in diabetic rats.	[31]
Leaf extract	Ethanol (95%)	—	Neuroprotective, antiapoptotic, and anti-amnesic effects. Mitigator of the toxicity by aluminum nanoparticles compared to the dopenzil standard drug (when induced Alzheimer’s disease in rats’ model).	[9]
Seed extract	Ethyl acetate	(R,S)-5-methoxy-8-[(6,7-dihydroxy-3,7-dimethyl-2-octenyl) oxy] psoralen, casimiroin and 5,6,2′-trimethoxyflavone.	Antimutagenic effects	[32]

**Table 2:** Abundant phytochemical peaks identified in CE extract via FI-ESI FTICR-MS analysis in positive electrospray ionization mode (ESI+).

*m*/*z*	Name	Molecular Formula	Peak Abundance *	% Relative Abundance *	Class	Subclass	Reported Therapeutic Properties
343.1182	Tetramethylscutellarein **	C19H18O6	3.59 × 10^8^	40.34	Polyphenol	Flavonoid	Antioxidant, anti-inflammatory, antibacterial, hemostatic, and wound-healing effects Modulator of bacterial drug resistance
633.1437	Rutin */**	C27H30O16	1.20 × 10^8^	13.53	Polyphenol	Flavonoid	Antioxidant, antibacterial, antibiofilm, wound-healing properties for normal and diabetic skin and for cold burn wounds, anti-inflammatory
343.0882	(S)-Usnate **	C18H14O7	3.59 × 10^7^	4.03	Organic compound	Dibenzofurano derivative	Antibacterial, immunostimulant, antiviral, antifungal, anti-inflammatory, antitumor, antimicrobial, and antiparasitic *
343.1295	Lactose **	C12H22O11	3.20 × 10^7^	3.60	Carbohydrate	disaccharide	Anti-aging agent
621.2367	Eugenol 2-O-Beta-D-Glucopyranosyl-(1-2)-(O-Beta-D-Xylopyranosyl-(1-6))-O-Beta-D-Glucopyranoside	C27H40O16	2.71 × 10^7^	3.05	Polyphenol	Flavonoid/Phenylpropanoid	Related to eugenol
395.1473	Rotenone	C23H22O6	2.39 × 10^7^	2.69	Polyphenol	Flavonoid/isoflavone	Insecticide, acaricide, pesticide
343.1016	Caffeic Acid 4-O-Glucoside **	C15H18O9	2.17 × 10^7^	2.44	Polyphenol	Flavonoid	Antioxidant, inhibitor of advanced glycation end products, inhibitor of the angiotensin-converting enzyme, and the acetylcholinesterase enzyme
593.2769	Protoporphyrin Ix	C34H32N4O4	2.03 × 10^7^	2.28	Organic compound	Porphyrin	Intermediate in the biosynthesis of heme, chlorophyll, and cobalamin, for diagnosis and cancer therapy
343.0967	Methionine-Betaxanthin **	C14H18N2O6S	1.77 × 10^7^	1.99	N-containing compound (Betalains)	Betaxanthins	Antimicrobial, antifungal, and anticancer
313.1135	Primeverose	C11H20O10	1.73 × 10^7^	1.94	Carbohydrate	Disaccharide	Related to primeverose derivatives
343.1394	Coniferin **	C16H22O8	1.72 × 10^7^	1.93	Organic compound (Phenolic glycoside)	Monosaccharide derivative	For rheumatoid arthritis, an anti-inflammatory, antioxidant, and mitochondrial transmembrane disrupter
343.1438	Cinnamoyl-glucose **	C15H18O7	1.51 × 10^7^	1.70	Polyphenol	Flavonoid/phenylpropanoid	Anti-glycation, antibacterial, antioxidant, and skin anti-aging properties
649.0927	Vicenin-2 **	C27H29O15	1.26 × 10^7^	1.42	Polyphenol	Flavonoid	Anti-glycation, antioxidant, anti-inflammatory, antiseptic, renal-protective, anticancer, trypanocidal, antispasmodic, anti-diabetic, and hepatoprotective, diabetic wound healing, and skin photoprotective properties
313.1016	Threonine-Betaxanthin **	C13H16N2O7	1.21 × 10^7^	1.36	N-containing compound (Betalains)	Betaxanthins	Antimicrobial, antifungal, and anticancer
343.1719	Magnoflorine **	C20H24NO4	1.10 × 10^7^	1.24	Alkaloid (Isoquinoline alkaloid)	Aporphine alkaloid	Anti-diabetic, antioxidant, anti-inflammatory, immunomodulatory, anticancer, relaxant, anti-osteoporotic, antibacterial, antifungal, antidepressant, sedative, neuroprotective, and anxiolytic
611.1994	Hesperidin */**	C28H34O15	1.00 × 10^7^	1.12	Polyphenol	Flavonoid	Antioxidant, anticancer, antibacterial, antifungal, anti-diabetic, antimalarial, neuroprotective, cardio-protective, anti-inflammatory
760.5886	1-Palmitoyl-2-Oleoyl-Phosphatidylcholine **	C42H82NO8P	9.84 × 10^6^	1.11	Lipid (phospholipid)	Diacylglycerol phospholipid	Enhances cognitive functions, protects heart reperfusion injury, and prevents irreversible cardiac damage
207.1073	Acetyleugenol **	C12H14O3	8.67 × 10^6^	0.97	Polyphenol	Phenylpropanoid	Antibacterial, anticancer, anti-inflammatory
611.1386	Prodelphinidindimer B3 **	C30H26O14	8.48 × 10^6^	0.95	Polyphenol	Flavonoid/Flavan-3-ols	Antioxidant, antibacterial, and anti-tumor
637.1767	Kaempferol3-O-(6-Acetyl-Galactoside)7-O-Rhamnoside **	C29H32O16	6.99 × 10^6^	0.79	Polyphenol	Flavonoid	Antioxidant, antibacterial, and antitumor
801.5554	1-18_2-2-18_2-Monogalactosyldiacylglycerol **	C45H78O10	6.87 × 10^6^	0.77	Lipid	Glycerolipid	Anti-inflammatory and cardioprotective properties
797.5179	1-18_1-2-16_3-Monogalactosyldiacylglycerol **	C43H74O10	6.70 × 10^6^	0.75	Lipid	Glycerolipid	Anti-inflammatory and cardioprotective properties
635.3905	3-O-Trans-P-Coumaroyltormentic Acid **	C39H54O7	5.80 × 10^6^	0.65	Terpene	Triterpenoid	Antifungal, antiviral, antimicrobial, anti-inflammatory, antiparasitic, antihyperglycemic, anticancer, and analgesic
344.1472	Sinapoyltyramine **	C19H21NO5	5.37 × 10^6^	0.60	N-containing compound	Cinnamic acids and derivatives	Antimicrobial and antifungal.
266.1135	Thiamine	C12H17N4OS	5.34 × 10^6^	0.60	Organic compound/Diazines	Pyrimidines and pyrimidine derivatives	Neuroprotective
774.5608	1-Palmitoyl-2-Vernoloyl-Phosphatidylcholine **	C42H80NO9P	4.70 × 10^6^	0.53	Lípid	Phosphatidylcholines	Anti-inflammatory and cardioprotective properties
199.2090	2-Tridecanone	C13H26O	3.39 × 10^6^	0.38	Organic compound (ketones)	Organooxygen compound	Insecticide
751.6339	Plastoquinol-9 **	C53H82O2	3.19 × 10^6^	0.36	Lipid	Polyprenyl quinols/Prenol lipids	Antioxidant
444.1663	5,6,7,8-Tetrahydrofolate *	C19H21N7O6	3.06 × 10^6^	0.34	N-containing compound	Pteridine derivative (tetrahydrofolic acid)	Cofactor
405.1165	Dehydro-Glucosyl-Piceatannol **	C20H20O9	3.00 × 10^6^	0.34	Polyphenol	Phenolic acid derivative (stilbenoid)	Antioxidant, antimicrobial, anti-diabetic, anti-inflammatory, antiproliferative, cardioprotective, and neuroprotective effects
320.1709	(Indol-3-Yl) Acetyl-L-Leucine **	C16H19N2O3	2.81 × 10^6^	0.32	Alkaloid	Indole alkaloid	Anti-inflammatory, antitumor, antibacterial, antifungal, and antiviral.
625.4091	Alphitolic Acid (3-O-Cis-P-Coumaroyl-) **	C39H54O6	2.70 × 10^6^	0.30	Terpene	Miscellaneous triterpenoid	Antifungal, antiviral, antimicrobial, anti-inflammatory, antiparasitic, antihyperglycemic, anticancer, and analgesic
423.1675	Scopoletin7-Glucoside **	C21H26O9	2.63 × 10^6^	0.30	Polyphenol	Flavonoid (Coumarins and/coumarin glycosides)	Antioxidant, anticancer, antibacterial, antifungal, anti-diabetic, antimalarial, neuroprotective, cardio-protective, anti-inflammatory
519.1912	Ciceritol **	C19H34O16	1.98 × 10^6^	0.22	Carbohydrate	Organooxygen compound (o-glycosyl compound)	Antioxidant, antitumor, immune-enhancing, antibacterial, anticoagulant, and hypoglycemic
361.1425	3-Methoxy-Tyramine-Betaxanthin **	C18H20N2O6	1.74 × 10^6^	0.20	N-containing compound (Betalains)	Betaxanthins	Antimicrobial, antifungal, and anticancer
365.0557	5-Formamido-1-(5-Phospho-D-Ribosyl)-Imidazole-4-Carboxamide	C10H13N4O9P	1.53 × 10^6^	0.17	N-containing compound	Nucleoside/nucleotide	Intermediate in purine metabolism
1318.3754	Cyanidin3-(Feruloyl) (Sinapoyl)-Triglucoside-5-Glucoside **	C60H69O33	1.47 × 10^6^	0.17	Polyphenol	Flavonoid	Antioxidant, anticancer, antibacterial, antifungal, anti-diabetic, antimalarial, neuroprotective, cardio-protective, anti-inflammatory
455.2071	Obacunone **	C26H30O7	1.46 × 10^6^	0.16	Terpene	Triterpenoid (Prenol lipid)	Antifungal, antiviral, antimicrobial, anti-inflammatory, antiparasitic, antihyperglycemic, anticancer, and analgesic
534.2548	Pyropheophorbide A **	C33H33N4O3	1.45 × 10^6^	0.16	N-containing compound	Tetrapyrroles and derivatives	Antioxidant, anti-inflammatory, and phototoxic and cytotoxic
285.1988	Hexadecanedioate **	C16H28O4	1.41 × 10^6^	0.16	Lipid	Fatty Acyls (long-chain fatty acids)	Anti-inflammatory and cardioprotective properties

* Total sum of peak abundance and % relative abundance of these compounds in the positive and negative ESI modes. ** The compounds relevant to wound healing were selected because they exhibited anti-inflammatory, antioxidant, antimicrobial (antibacterial), antibiotic, and antifungal activities in previous studies, and other effects that aid the wound-healing process.

**Table 3:** Abundant phytochemical peaks identified in CE extract via FI-ESI FTICR-MS analysis in negative electrospray ionization mode (ESI−).

*m*/*z*	Name	Molecular Formula	Peak Abundance *	% Relative Abundance *	Class	Subclass	Reported Therapeutic Properties
645.1254	Rutin */**	C27H30O16	1.20 × 10^8^	13.53	Polyphenol	Flavonoid	Antioxidant, antibacterial, antibiofilm, wound-healing properties for normal and diabetic skin and for cold-burn wounds, anti-inflammatory.
609.1857	Hesperidin */**	C28H34O15	1.00 × 10^7^	1.12	Polyphenol	Flavonoid	Antioxidant, anticancer, antibacterial, antifungal, anti-diabetic, antimalarial, neuroprotective, cardio-protective, anti-inflammatory.
609.1084	Quercetin3-O-Xylosyl-Glucuronide **	C26H26O17	4.63 × 10^6^	0.52	Polyphenol	Flavonoid	Antioxidant, anticancer, antibacterial, antifungal, anti-diabetic, antimalarial, neuroprotective, cardio-protective, anti-inflammatory.
351.131	Kahweol **	C20H26O3	2.74 × 10^6^	0.31	Terpene	Diterpene	Antifungal, antiviral, antimicrobial, anti-inflammatory, antiparasitic, antihyperglycemic, anticancer, and analgesic.
215.0504	Syringic Acid **	C9H10O5	2.54 × 10^6^	0.29	Polyphenol	Phenolic acid	Antioxidant, antimicrobial, anticancer, anti-inflammatory, anti-mutagenic.
533.1738	3-Isobutanoyl-4-(3-Methylbutanoyl) Sucrose **	C21H36O13	2.46 × 10^6^	0.28	Carbohydrate	Disaccharide	Immunostimulant, anti-inflammatory, and anti-apoptotic associated effects.
555.2869	Villanovanei **	C26H40O9	2.04 × 10^6^	0.23	Terpene	Diterpene (diterpenoid glucoside)	Antifungal, antiviral, antimicrobial, anti-inflammatory, antiparasitic, antihyperglycemic, anticancer, and analgesic.
421.1655	Hydroxy-P-Menthan-7-Oic Acid glucuronide **	C16H26O9	1.87 × 10^6^	0.21	Terpene	Monoterpenoid	Antifungal, antiviral, antimicrobial, anti-inflammatory, antiparasitic, antihyperglycemic, anticancer, and analgesic.
311.078	1-O-Vanilloyl-Beta-D-Glucose **	C14H18O9	1.53 × 10^6^	0.17	Polyphenol	Phenolic acids	Antioxidant, antitumor, immune-enhancing, antibacterial, anticoagulant, and hypoglycemic.
625.4194	8-(3,4-Dihydroxy-5-Alkenyl) Phenyl-3-(9e,11e,13z-Pentadecatrienyl) Catechol **	C42H58O4	1.33 × 10^6^	0.15	Lipid	Phenolic lipid	Antioxidant, antimicrobial, anticancer, anti-inflammatory, anti-mutagenic.
311.1693	Quinamine **	C19H24N2O2	1.28 × 10^6^	0.14	Alkaloid	Isoquinoline	Anti-inflammatory, antitumor, antibacterial, antifungal, and antiviral.
609.3252	26-Hydroxybrassinolide	C28H48O7	1.24 × 10^6^	0.14	Lipids and lipid-like molecules	Steroid Brassinosteroids	Immune-enhancing y for metabolic disorders
499.0656	Rhaponticin **	C21H24O9	1.21 × 10^6^	0.14	Lipid	Glycerolipid	Anti-inflammatory and cardioprotective properties.
305.0755	Glutathione *	C10H16N3O6S	9.82 × 10^5^	0.11	N-containing compound	Carboxylic acids and derivatives (tripeptide)	Antioxidant
963.2426	Patuletin3-O-(2-Feruloylglucosyl) (1-6)-(Apiosyl(1-2))-Glucoside **	C43H48O25	9.79 × 10^5^	0.11	Polyphenol	Flavonoid	Antioxidant, anticancer, antibacterial, antifungal, anti-diabetic, antimalarial, neuroprotective, cardio-protective, anti-inflammatory.
451.025	Glucoerysolin **	C12H22NO11S3	9.61 × 10^5^	0.11	Carbohydrate	Monosaccharide	Antioxidant, antitumor, immune-enhancing, antibacterial, anticoagulant, and hypoglycemic.
415.1376	Conidendrin **	C20H20O6	8.43 × 10^5^	0.09	Polyphenol	Lignan lactone	Antioxidant and anti-inflammatory, anti-menopausal, and antitumor.
630.1388	Peonidin3-O-(6-P-Coumaroyl-Glucoside) **	C31H29O13	7.49 × 10^5^	0.08	Polyphenol	Flavonoid (phenylpropanoid)	Antioxidant, anticancer, antibacterial, antifungal, anti-diabetic, antimalarial, neuroprotective, cardio-protective, anti-inflammatory.
441.2138	9-F1-Phytoprostane	C18H32O5	6.98 × 10^5^	0.08	Lipid	Phytoprostane (prostanoid)	Immune-enhancing y for metabolic disorders.
455.0889	A Reduced Flavodoxin	C17H21N4O9P	6.60 × 10^5^	0.07	Proteins	Amino acid sequences	Cofactor
366.1513	Arginine-Betaxanthin **	C15H21N5O6	6.49 × 10^5^	0.07	N-containing compounds (Betalains)	Betaxanthins	Antimicrobial, antifungal, and anticancer.
697.4853	Dioleoyl Phosphatidate **	C39H71O8P	6.36 × 10^5^	0.07	Lipid	Phosphatidate (phospholipid)	Anti-inflammatory and cardioprotective properties.
315.0747	Protocatechuic Acid4-O-Glucoside **	C13H16O9	6.18 × 10^5^	0.07	Carbohydrate	Phenolic glycosides	Antioxidant, antitumor, immune-enhancing, antibacterial, anticoagulant, and hypoglycemic.
293.0502	5-Amino-1-(5-Phospho-Beta-D-Ribosyl) Imidazole	C8H13N3O7P	5.62 × 10^5^	0.06	N-containing compound	(Nucleosides, nucleotides) 1-(Phosphoribosyl) imidazoles	Intermediate in the biosynthesis of purines and thiamine.
336.0822	Trans-5-O-(4-Coumaroyl)-D-Quinate	C16H17O8	5.57 × 10^5^	0.06	Organic compound (organic oxygen compound)	Quinic acids and derivatives	Cofactor
503.1402	17-Decarboxy-Neobetanin **	C23H24N2O11	5.55 × 10^5^	0.06	Carbohydrate	O-glycosyl compounds	Antioxidant, antitumor, immune-enhancing, antibacterial, anticoagulant, and hypoglycemic.
542.2025	Trans-Zeatin-O-Glucoside-7-N-Glucoside **	C22H33N5O11	5.52 × 10^5^	0.06	Organic compound	Cytokinin conjugates	Antioxidant, antitumor, immune-enhancing, antibacterial, anticoagulant, and hypoglycemic.
305.0853	Convicine	C10H15N3O8	5.45 × 10^5^	0.06	N-containing compound	Pyrimidine glycoside	Anti-nutritional factor.
67.05447	Isoprene **	C5H8	5.43 × 10^5^	0.06	Lipid	Prenol Lipids (Isoprenoids)	Anti-inflammatory and cardioprotective properties.
442.1565	5,6,7,8-Tetrahydrofolate	C19H21N7O6	3.06 × 10^6^	0.34	N-containing compound	Pteridine derivative (tetrahydro folic acid)	Cofactor
576.392	Tomatidine Galactoside	C33H55NO7	5.29 × 10^5^	0.06	Lipid	Sterol Lipids	Immune-enhancing y for metabolic disorders.
103.0271	Nicotinamide **	C6H6N2O	5.19 × 10^5^	0.06	Alkaloid	Pyridine alkaloids (Nicotinic acid alkaloids)	Anti-inflammatory, antitumor, antibacterial, antifungal, and antiviral.
124.9656	Dimethyltrisulfide **	C2H6S3	5.07 × 10^5^	0.06	Organosulfur compound	Organosulfur compounds (organic trisulfide)	Antifungal and for biocontrol.
533.2042	Phillyrin **	C27H34O11	5.02 × 10^5^	0.06	Polyphenol	Lignans, neolignanes (lignan glycosides)	Antioxidant and anti-inflammatory, anti-menopausal, and antitumor.

* Total sum of peak abundance and % relative abundance of these compounds in the positive and negative ESI modes. ** The compounds relevant to wound healing were selected because they exhibited anti-inflammatory, antioxidant, antimicrobial (antibacterial), antibiotic, and antifungal activities in previous studies, and other effects that aid the wound-healing process.

**Table 4:** Characterization of CE-NP (n = 3).

Batch	OP (v:v)	Extraction Solvent/CE (mg)	Size ± SD (nm)	PdI ± SD	Z Potential ± SD (mV)	pH ± SD	Comments
1	ACE: EtAc (50:50)	hexane/5	311.567	0.218	0.455	--	Visual physical instability
2	ACE: EtAc (50:50)	hexane/50	423.767	0.356	−2.783	--	Visual physical instability
3	ACE: EtAc (50:50)	hexane/100	715.601	0.638	−3.663	--	Visual physical instability
4	ACE: EtAc (50:50)	Et/5	-	-	-	6.427 ± 0.15	Visual physical instability
5	ACE: EtAc (50:50)	Et/7.5	-	-	-	6.733 ± 0.14	Visual physical instability
6	ACE: EtAc (50:50)	Et/10	-	-	-	6.903 ± 0.05	Visual physical instability
7	ACE: MEK (75:25)	Et/10	169.30 ± 1.30	0.08 ± 0.03	−9.45 ± 1.73	6.47 ± 0.12	Good visual and physical stability
8	ACE: MEK (75:25)	EtAc/10	171.37 ± 2.35	0.08 ± 0.01	−12.57 ± 1.42	6.73 ± 0.11	Few visual and physical instabilities
9	ACE: MEK (75:25)	n-hexane/10	177.97 ± 1.20	0.07 ± 0.03	−10.45 ± 0.66	6.07 ± 0.05	Few visual and physical instabilities
10 (Placebo)	ACE: MEK (75:25)	Et/10	166.90 ± 0.96	0.11 ± 0.03	−10.10 ± 0.78	6.11 ± 0.04	Good visual and physical stability

OP: organic phase; ACE: acetone; EtAc: ethyl acetate; MEK: methyl ethyl ketone; Et: ethanol; CE: *Casimiroa edulis* extract; PdI: polydispersity index; SD: standard deviation.

## Data Availability

The original contributions presented in this study are included in the article/Supplementary Materials. Further inquiries can be directed to the corresponding author(s).

## Supplementary Materials

The following supporting information can be downloaded at: https://www.mdpi.com/article/10.3390/pharmaceutics18020249/s1. Figure S1. CE-NP formulation using 10 mg of ethanolic extract, prepared by the rapid emulsion-diffusion method. Table S1. Abundant phytochemical peaks identified in CE extract via FI-ESI FTICR-MS analysis in positive electrospray ionization mode (ESI+, <100 mDa). Table S2. Abundant phytochemical peaks identified in CE extract via FI-ESI FTICR-MS analysis in negative electrospray ionization mode (ESI−, <100 mDa).

## Abbreviations

The following abbreviations are used in this manuscript:

ACE	Acetone
AgNPs	Silver nanoparticles
ANOVA	Analysis of variance
BS	Backscattering
CE	*Casimiroa edulis* La Llave et Lex
CE-NP	*Casimiroa edulis*-loaded nanoparticles
DI-ESI-FT-ICR-MS	Direct-infusion electrospray ionization Fourier transform ion cyclotron resonance mass spectrometry
DPPH	2, 2-diphenyl 1-picryl hydrazyl
Et	Ethanol
EtAc	Ethyl acetate
ESI−	Negative electrospray ionization mode
ESI+	Positive electrospray ionization mode
FIA-ESI-FTICR-MS	Direct injection ion cyclotron Fourier transform mass spectrometry
HeLa	Cervical adenocarcinoma
MeOH	Methanol
MEK	Methyl ethyl ketone
NP-Placebo	Placebo nanoparticle
OF	Occlusion factor
OP	Organic phase
PBS	Phosphate-buffered saline
PLGA	Poly (DL-lactide-co-glycolide) acid terminated
PdI	Polydispersity index
PSAT1	Phosphoserine aminotransferase 1
PVA	Polyvinyl alcohol 4-88
SD	Standard deviation
TEM	Transmission electron microscopy
TiO_2_ NPs	Titanium dioxide nanoparticles
UV–Vis	Ultraviolet–visible
ZnO NPs	Zinc oxide nanoparticles
