# Supplementary material for: Casimiroa edulis Leaf Extract–Loaded PLGA Nanoparticles: Untargeted Phytochemical Profiling and Wound-Healing-Oriented Antioxidant/Occlusive Characterization
Source: Pharmaceutics. 2026 Feb 17;18(2):249. doi: 10.3390/pharmaceutics18020249 (PMC12944296; doi:10.3390/pharmaceutics18020249)
Supplement: Supplementary file 1 [file pharmaceutics-18-00249-s001.zip › pharmaceutics-4140657-supplementary.pdf]

Supplementary Materials: *Casimiroa edulis* Leaf Extract–Loaded PLGA Nanoparticles: Untargeted Phytochemical Profiling and Wound-Healing-Oriented Antioxidant/Occlusive Characterization

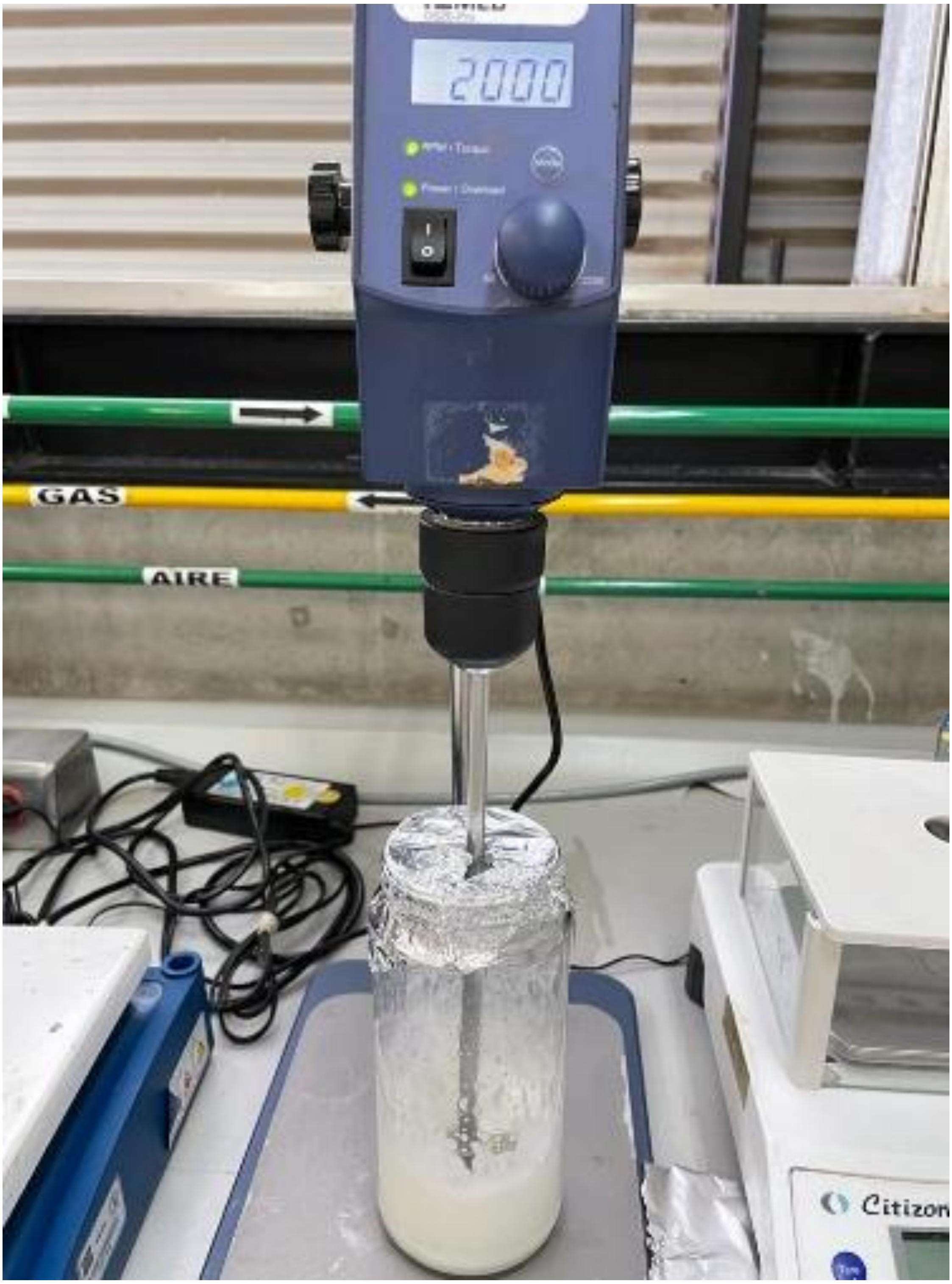

Figure S1. CE-NP formulation using 10 mg of ethanolic extract, prepared by the rapid emulsion-diffusion method.

Table S1. Abundant phytochemicals peaks identified in CE extract via FI-ESI FTICR-MS analysis in positive electrospray ionization mode (ESI+, <100 mDa).

m/z	NAME	MOLECULAR FORMULA	PEAK ABUNDANCE	% RELATIVE ABUNDANCE
343.11093	Tetramethylscutellarein	C19H18O6	8.05E+08	26.96%
621.30795	Eugenol 2-O-Beta-D-Glucopyranosyl-(1 - 2)-(O-Beta-D-Xylopyranosyl-(1 - 6))-O-Beta-D-Glucopyranoside	C27H40O16	6.37E+08	21.33%
343.12541	Cinnamoylglucose	C15H18O7	8.63E+07	2.89%
343.08819	(S)-Usnate	C18H14O7	7.77E+07	2.60%
633.14374	Rutin	C27H30O16	7.08E+07	2.37%
395.14728	Rotenone	C23H22O6	5.46E+07	1.83%
637.24839	2-Acetyl-3-(2-Methylbutanoyl)-3,4-Di(3-Methylbutanoyl)Sucrose	C29H48O15	5.44E+07	1.82%
677.20012	Alpha-Hydroxyheme	C34H30FeN4O5	4.35E+07	1.46%
207.11162	Acetyleugenol	C12H14O3	4.29E+07	1.44%
633.1676	Vescalin	C27H20O18	4.28E+07	1.43%
635.3905	3-O-Trans-P-Coumaroyltormentic Acid	C39H54O7	4.16E+07	1.39%
313.11353	Primeverose	C11H20O10	4.08E+07	1.36%
365.05573	5-Formamido-1-(5-Phospho-D-Ribosyl)-Imidazole-4-Carboxamide	C10H13N4O9P	3.67E+07	1.23%
343.17191	Magnoflorine	C20H24NO4	3.28E+07	1.10%
343.12948	Lactose	C12H22O11	3.20E+07	1.07%
313.10157	Threonine-Betaxanthin	C13H16N2O7	2.94E+07	0.98%
593.27693	Cucurbitacin C	C32H48O8	2.91E+07	0.98%
625.40911	Alphitolic Acid (3-O-Cis-P-Coumaroyl-)	C39H54O6	2.89E+07	0.97%
811.55814	1-18_1-2-16_3-Monogalactosyldiacylglycerol	C43H74O10	2.86E+07	0.96%
343.15345	Coniferin	C16H22O8	2.70E+07	0.90%
886.59441	3-Methoxy-4-Hydroxy-5-All-Trans-Decaprenylbenzoate	C58H87O4	2.58E+07	0.86%
707.18102	Epicatechin-(2a-7)(4a-8)-Epicatechin 3-O-Galactoside	C36H34O15	2.58E+07	0.86%
611.1994	Hesperidin	C28H34O15	2.55E+07	0.85%
423.1786	Scopoletin7-Glucoside	C21H26O9	2.49E+07	0.83%
343.10156	Caffeic Acid 4-O-Glucoside	C15H18O9	2.17E+07	0.73%
343.18121	(S)-Magnoflorine	C20H24NO4	2.13E+07	0.71%
593.27693	Protoporphyrin Ix	C34H32N4O4	2.03E+07	0.68%
653.56138	Stigmastanol Ferulate	C39H60O4	1.96E+07	0.66%
653.3597	Bis(Beta-D-Glucosyl) Crocetin	C32H44O14	1.94E+07	0.65%
873.60632	3-Demethylubiquinol-10	C58H90O4	1.82E+07	0.61%
343.09674	Methionine-Betaxanthin	C14H18N2O6S	1.77E+07	0.59%
218.24225	1-Methyl-2-Pentyl-1,2,3,4-Tetrahydroquinoline	C15H23N	1.58E+07	0.53%
344.13851	Sinapoyltyramine	C19H21NO5	1.50E+07	0.50%
519.1748	Ciceritol	C19H34O16	1.45E+07	0.49%
707.49135	Beta-Chaconine	C39H64NO10	1.44E+07	0.48%
381.08953	Quercetin 3-Sulfate	C15H8O10S	1.38E+07	0.46%
801.5554	1-18_2-2-18_2-Monogalactosyldiacylglycerol	C45H78O10	1.37E+07	0.46%
298.30866	Ricinoleate	C18H33O3	1.35E+07	0.45%
649.09267	Vicenin-2	C27H29O15	1.26E+07	0.42%
611.13864	Quercetin3-O-Xylosyl-Glucuronide	C26H26O17	1.22E+07	0.41%

707.35053	2-Acetyl-3-Decanoyl-3,4-Di(3-Methylbutanoyl)Sucrose	C34H58O15	1.05E+07	0.35%
481.21914	Atractyligenin(2-O-Beta-Glucopyranosyl-)	C26H40O8	1.05E+07	0.35%
760.58863	1-Palmitoyl-2-Oleoyl-Phosphatidylcholine	C42H82NO8P	9.84E+06	0.33%
752.51343	2-Acetyl-3-Dodecanoyl-3,4-Di(3-Methylbutanoyl)Sucrose	C36H62O15	9.47E+06	0.32%
593.30341	Pheophorbidea	C35H36N4O5	8.80E+06	0.29%
377.13665	Todolactola	C20H24O7	8.75E+06	0.29%
649.72308	A Diphospho-1d-Myo-Inositol Tetrakisphosphate	C6H7O24P6	8.63E+06	0.29%
611.13864	Prodelphinidindimer B3	C30H26O14	8.48E+06	0.28%
320.1709	(Indol-3-Yl)Acetyl-L-Leucine	C16H19N2O3	8.42E+06	0.28%
411.13121	Lactupicrin	C23H22O7	7.91E+06	0.26%
915.68939	1-16_0-2-18_3-Digalactosyldiacylglycerol	C49H86O15	7.37E+06	0.25%
637.1767	Kaempferol3-O-(6-Acetyl-Galactoside)7-O-Rhamnoside	C29H32O16	6.99E+06	0.23%
312.31516	9,10-12,13-Diepoxyoctadecanoate	C18H31O4	6.58E+06	0.22%
679.28302	Rheumlhasola	C43H34O8	6.54E+06	0.22%
381.06544	Asparagine-Betaxanthin	C13H15N3O7	6.36E+06	0.21%
633.21518	Cofaryloside I	C33H44O12	6.22E+06	0.21%
343.22163	5-Heptadecatrienylresorcinol	C23H34O2	6.09E+06	0.20%
108.95233	1,2-Benzoquinone	C6H4O2	5.99E+06	0.20%
414.20416	N6-(Delta2-Isopentenyl)-Adenosine 5-Monophosphate	C15H20N5O7P	5.71E+06	0.19%
459.30908	Demethylphylloquinol	C30H46O2	5.54E+06	0.19%
266.11352	Thiamine	C12H17N4OS	5.34E+06	0.18%
652.44394	All-Trans-Heptaprenyl Diphosphate	C35H57O7P2	5.33E+06	0.18%
342.14011	Dihydropapaverine	C20H23NO4	5.24E+06	0.18%
397.06913	Littorine	C17H24NO3	5.19E+06	0.17%
351.08461	Corydamine	C20H18N2O4	4.92E+06	0.16%
774.56076	1-Palmitoyl-2-Vernoloyl-Phosphatidylcholine	C42H80NO9P	4.70E+06	0.16%
679.37893	3-Dodecanoyl-3-Isobutanoyl-4-(3-Methylbutanoyl)Sucrose	C33H58O14	4.59E+06	0.15%
649.57942	Menaquinone-7	C46H64O2	4.22E+06	0.14%
469.16322	Humulone	C21H30O5	4.19E+06	0.14%
393.29802	Lathosterol	C27H46O	4.16E+06	0.14%
805.76544	6-(All-Trans-Decaprenyl)-2-Methoxy-Phenol	C57H88O2	4.16E+06	0.14%
430.17846	Cis-Zeatin Riboside Monophosphate	C15H20N5O8P	4.07E+06	0.14%
625.35212	Red Chlorophyll Catabolite	C35H36N4O7	3.96E+06	0.13%
1674.08187	N-Acetyl-Beta-D-Glucosaminyl-(1-4)-N-Acetyl-Alpha-D-Glucosaminyl-Diphosphodolichol	C96H158N2O17P2	3.80E+06	0.13%
595.47101	Caffarolidea	C35H56O6	3.55E+06	0.12%
125.13548	Guaiacol	C7H8O2	3.49E+06	0.12%
853.61395	Alpha-Chaconine	C45H74NO14	3.43E+06	0.11%
1719.1058	Lambertianinb	C75H50O48	3.40E+06	0.11%
109.12077	1 2-Dimethylpyridinium	C7H10N	3.40E+06	0.11%
199.20896	2-Tridecanone	C13H26O	3.39E+06	0.11%
313.08263	2-(Formamido)-N1-(5-Phospho-Beta-D-Ribosyl)Acetamidine	C8H15N3O8P	3.35E+06	0.11%
659.34703	Fucoxanthin	C42H58O6	3.34E+06	0.11%

515.14786	Bixin Dimethyl Ester	C26H32O4	3.31E+06	0.11%
573.17482	Biotinyl-5-Adenylate	C20H27N7O9PS	3.29E+06	0.11%
211.05721	D-Glucaric Acid	C6H10O8	3.24E+06	0.11%
722.50805	Beta-Solanine	C39H63NO11	3.22E+06	0.11%
751.63393	Plastoquinol-9	C53H82O2	3.19E+06	0.11%
1050.33575	Juniperonoyl-Coa	C41H62N7O17P3S	3.19E+06	0.11%
503.32012	Medicagenic Acid	C30H46O6	3.18E+06	0.11%
651.28369	Limoninglucoside	C32H42O14	3.04E+06	0.10%
405.11652	Dehydro-Glucosyl-Piceatannol	C20H20O9	3.00E+06	0.10%
595.47101	Caffarolidef	C37H54O6	2.83E+06	0.09%
693.1994	Curcumin Diglucoside	C33H40O16	2.77E+06	0.09%
325.04846	Erucinn-Acetyl-Cysteine	C11H20N2O3S3	2.75E+06	0.09%
755.54088	1-18_2-2-16_0-Monogalactosyldiacylglycerol	C43H78O10	2.73E+06	0.09%
625.22764	Verbascoside	C29H36O15	2.67E+06	0.09%
383.20436	(3s,5r,6r)-3,5-Dihydroxy-6,7-Didehydro-5,6-Dihydro-12-Apo-Beta-Caroten-12-Al	C25H34O3	2.67E+06	0.09%
728.70589	1d-Myo-Inositol 3-Diphosphate 1,2,4,5,6-Pentakisphosphate	C6H7O27P7	2.64E+06	0.09%
537.37164	B-Carotene	C40H56	2.61E+06	0.09%
453.11607	Aspalathin	C21H24O11	2.60E+06	0.09%
541.24371	Oleuropein	C25H32O13	2.59E+06	0.09%
695.30926	1 2-Diferuloylgentiobiose	C32H38O17	2.55E+06	0.09%
591.33939	Pheophorbide A	C35H34N4O5	2.55E+06	0.09%
444.16633	5,6,7,8-Tetrahydrofolate	C19H21N7O6	2.52E+06	0.08%
717.24455	Theaflavin3-O-Gallate	C36H28O16	2.49E+06	0.08%
319.36154	Phytosphingosine (C18)	C18H40NO3	2.42E+06	0.08%
609.45045	Caffarolideg	C38H56O6	2.41E+06	0.08%
434.19374	Pelargonidin-3-O-Galactoside	C21H21O10	2.37E+06	0.08%
522.20627	Petunidin3-O-(6-Acetyl-Galactoside)	C24H25O13	2.34E+06	0.08%
774.26017	Cyanidin3-O-Diglucoside-5-O-Glucoside	C33H41O21	2.31E+06	0.08%
525.26651	4-Dodecanoylsucrose	C24H44O12	2.27E+06	0.08%
1836.23036	Beta-D-Man-(1-4)-Beta-D-Glcnac-(1-4)-Alpha-D-Glcnac-Diphosphodolichol	C102H168N2O22P2	2.21E+06	0.07%
507.2571	2-Decarboxy-Neobetanin	C23H26N2O11	2.14E+06	0.07%
503.18662	6-O-Malonyldaidzin	C24H22O12	2.14E+06	0.07%
551.50646	Anhydrolutein I	C40H54O	2.10E+06	0.07%
174.20562	L-Argininate	C6H13N4O2	2.07E+06	0.07%
393.10108	Macarpine	C22H18NO6	2.06E+06	0.07%
525.24761	(+)-Secoisolariciresinol Monoglucoside	C26H36O11	2.04E+06	0.07%
523.23161	3,7,4-Trimethylquercetin 2-O-Beta-D-Glucoside	C24H26O13	2.03E+06	0.07%
391.10094	Dopa-Betaxanthin	C18H18N2O8	2.00E+06	0.07%
529.19907	Malvidin3-O-Galactoside	C23H25ClO12	1.97E+06	0.07%
130.94072	Trichloroethene	C2HCl3	1.96E+06	0.07%
439.12548	Glucocheirolin	C11H20NO11S3	1.96E+06	0.07%
454.18938	Phytyl Diphosphate	C20H39O7P2	1.91E+06	0.06%
172.06087	3-Dehydroshikimate	C7H7O5	1.79E+06	0.06%
211.23152	Cyclo(Pro-Leu)	C11H18N2O2	1.77E+06	0.06%
553.38541	Kahweolpalmitate	C36H56O4	1.76E+06	0.06%
414.94336	D-Myo-Inositol (1,3,4)-Trisphosphate	C6H9O15P3	1.75E+06	0.06%
361.14246	3-Methoxy-Tyramine-Betaxanthin	C18H20N2O6	1.74E+06	0.06%

428.1723	R-Vicianin	C19H25NO10	1.65E+06	0.06%
631.12624	Pelargonidin3 5-O-Diglucoside	C27H31ClO15	1.61E+06	0.05%
497.36338	26-Hydroxybrassinolide	C28H48O7	1.60E+06	0.05%
617.25925	Pallidol3-O-Glucoside	C34H32O11	1.52E+06	0.05%
290.13939	L-Arginino-Succinate	C10H17N4O6	1.51E+06	0.05%
294.1499	Colneleate	C18H29O3	1.50E+06	0.05%
415.44247	Sitosterol	C29H50O	1.49E+06	0.05%
607.18138	Epoxypheophorbide A	C35H34N4O6	1.49E+06	0.05%
1318.3754	Cyanidin3-(Feruloyl)(Sinapoyl)-Triglucoside-5-Glucoside	C60H69O33	1.47E+06	0.05%
455.20714	Obacunone	C26H30O7	1.46E+06	0.05%
534.25482	Pyropheophorbide A	C33H33N4O3	1.45E+06	0.05%
285.19881	Hexadecanedioate	C16H28O4	1.41E+06	0.05%

Table S2. Abundant phytochemicals peaks identified in CE extract via FI-ESI FTICR-MS analysis in negative electrospray ionization mode (ESI-, <100 mDa).

m/z	NAME	MOLECULAR FORMULA	PEAK ABUNDANCE	% RELATIVE ABUNDANCE
645.12537	Rutin	C27H30O16	4.96E+07	18.76%
645.12537	Prodelphinidindimer B3	C30H26O14	4.96E+07	18.75%
625.50417	8-(3,4-Dihydroxy-5-Alkenyl)Phenyl-3-(9e,11e,13z-Pentadecatrienyl)Catechol	C42H58O4	1.83E+07	6.91%
609.17095	Hesperidin	C28H34O15	1.73E+07	6.55%
609.10843	Quercetin3-O-Xylosyl-Glucuronide	C26H26O17	9.98E+06	3.77%
455.08888	A Reduced Flavodoxin	C17H21N4O9P	4.61E+06	1.74%
313.19592	Kahweol	C20H26O3	3.54E+06	1.34%
629.47317	Caffarolideg	C38H56O6	3.39E+06	1.28%
495.15247	3-Isobutanoyl-4-(3-Methylbutanoyl)Sucrose	C21H36O13	2.96E+06	1.12%
593.15396	Biotinyl-5-Adenylate	C20H27N7O9PS	2.93E+06	1.11%
487.43006	4alpha-Hydroxymethyl-Ergosta-7,24(241)-Dien-3beta-Ol	C29H48O2	2.91E+06	1.10%
850.62941	3-Demethylubiquinol-10	C58H90O4	2.81E+06	1.06%
351.13103	Cannabidiol	C21H30O2	2.74E+06	1.04%
215.05039	Syringic Acid	C9H10O5	2.54E+06	0.96%
203.05714	Tryptophan	C11H12N2O2	2.50E+06	0.94%
442.10343	5,6,7,8-Tetrahydrofolate	C19H21N7O6	2.25E+06	0.85%
560.33819	16-Feruloyloxypalmitate	C26H39O6	2.11E+06	0.80%
555.28692	Villanovanei	C26H40O9	2.04E+06	0.77%
451.21196	Limonin	C26H30O8	2.04E+06	0.77%
377.10146	Oleuropein-Aglycone	C19H22O8	1.98E+06	0.75%
5.33E+02	Phillyrin	C27H34O11	1.91E+06	0.72%
421.16549	Hydroxy-P-Menthan-7-Oic Acidglucuronide	C16H26O9	1.87E+06	0.71%
490.20977	Cyanidin3-O-(6-Acetyl-Glucoside)	C23H23O12	1.71E+06	0.65%
539.13881	Oleuropein	C25H32O13	1.67E+06	0.63%
428.14203	Cis-Zeatin Riboside Monophosphate	C15H20N5O8P	1.60E+06	0.60%
311.078	1-O-Vanilloyl-Beta-D-Glucose	C14H18O9	1.53E+06	0.58%
453.2286	Obacunone	C26H30O7	1.33E+06	0.50%
323.06389	(S)-Stylopine	C19H17NO4	1.29E+06	0.49%

311.16926	Quinamine	C19H24N2O2	1.28E+06	0.48%
609.32523	26-Hydroxybrassinolide	C28H48O7	1.24E+06	0.47%
499.06559	Rhaponticin	C21H24O9	1.21E+06	0.46%
549.3196	Hordatinea	C28H38N8O4	1.13E+06	0.43%
323.07355	Erucinn-Acetyl-Cysteine	C11H20N2O3S3	1.09E+06	0.41%
935.69125	1-18_1-2-16_0-Digalactosyldiacylglycerol	C49H90O15	1.08E+06	0.41%
649.41703	3-Acetyl-3-Dodecanoyl-4-(3-Methylbutanoyl)Sucrose	C31H54O14	1.08E+06	0.41%
124.9842	Dimethyltrisulfide	C2H6S3	1.08E+06	0.41%
379.1644	Norbixin	C24H28O4	1.07E+06	0.41%
723.19957	1-Sinapoyl-2-Feruloylgentiobiose	C33H40O18	1.06E+06	0.40%
404.10586	2-(2,8-Dihydroxytridecyl)-6-Oxopyran-4-Olate	C18H29O5	1.05E+06	0.40%
542.37369	Castasterone 22-O-Sulfate	C28H47O8S	1.04E+06	0.39%
95.12171	Propan-1-Ol	C3H8O	1.03E+06	0.39%
425.07126	Archangelicin	C24H26O7	1.00E+06	0.38%
557.21185	Secoisolariciresinol-Sesquilignan	C30H38O10	9.99E+05	0.38%
305.07545	Glutathione	C10H16N3O6S	9.82E+05	0.37%
963.24259	Patuletin3-O-(2-Feruloylglucosyl)(1-6)-(Apiosyl(1-2))-Glucoside	C43H48O25	9.79E+05	0.37%
617.43959	Cafestololeate	C38H60O4	9.79E+05	0.37%
451.02501	Glucoerysolin	C12H22NO11S3	9.61E+05	0.36%
489.42659	(3beta,9beta)-4alpha-Demethyl-4alpha-Methylhydroxy-9,19-Cyclolanost-3-Ol	C30H52O2	9.40E+05	0.36%
848.19602	(S)-3-Hydroxy-Isobutanoyl-Coa	C25H38N7O18P3S	9.31E+05	0.35%
607.21087	Methylsalicylate 2-O-Beta-D-Glucopyranosyl-(1 - 2)-(O-Beta-D-Xylopyranosyl-(1 - 6))-O-Beta-D-Glucopyranoside	C25H36O17	9.13E+05	0.34%
408.18035	Linustatin	C16H27NO11	8.86E+05	0.33%
379.07491	Quercetin 3-Sulfate	C15H8O10S	8.60E+05	0.32%
363.19446	9-D1-Phytoprostane	C18H30O5	8.56E+05	0.32%
415.13755	Conidendrin	C20H20O6	8.43E+05	0.32%
557.24675	Carpaine	C28H50N2O4	8.35E+05	0.32%
459.39787	Protopanaxadiol	C30H52O3	8.22E+05	0.31%
509.11103	Daidzein-7-Glucuronide-4-Sulfate	C21H18O13S	7.96E+05	0.30%
483.20071	Cis-Tuberonic Acid Glucoside	C18H27O9	7.74E+05	0.29%
630.13883	Peonidin3-O-(6-P-Coumaroyl-Glucoside)	C31H29O13	7.49E+05	0.28%
1154.41125	3-Oxocerotoyl-Coa	C47H80N7O18P3S	7.46E+05	0.28%
602.4712	Ursolic Acid	C39H54O5	7.45E+05	0.28%
715.17882	Theaflavin3-O-Gallate	C36H28O16	7.39E+05	0.28%
479.16309	Myricetin 3-O-Galactoside	C21H20O13	7.29E+05	0.28%
521.15392	Cirsimarin	C23H24O11	7.11E+05	0.27%
491.14455	Isorhamnetin3-O-Glucuronide	C22H20O13	7.06E+05	0.27%
661.13774	Verbascoside	C29H36O15	6.98E+05	0.26%
441.21384	9-F1-Phytoprostane	C18H32O5	6.98E+05	0.26%
595.28702	Astaxanthin	C40H52O4	6.83E+05	0.26%
557.1479	Chamaemeloside	C27H28O14	6.73E+05	0.25%
445.10129	Sanguinarine	C20H14NO4	6.70E+05	0.25%

780.96071	Punicalin	C34H22O22	6.67E+05	0.25%
520.36261	2-C22_0-Dca-Lpa	C25H46O9P	6.65E+05	0.25%
647.52842	Menaquinone-7	C46H64O2	6.62E+05	0.25%
637.19043	Luteolin7-O-Diglucuronide	C27H26O18	6.62E+05	0.25%
819.6017	All Trans-Decaprenyl-2-Methoxy-6-1,4-Benzoquinol	C57H88O3	6.62E+05	0.25%
975.29835	Crocin	C44H64O24	6.61E+05	0.25%
366.15134	Arginine-Betaxanthin	C15H21N5O6	6.49E+05	0.25%
613.05878	Protoheme	C34H30FeN4O4	6.43E+05	0.24%
913.65527	1-16_0-2-18_3-Digalactosyldiacylglycerol	C49H86O15	6.37E+05	0.24%
833.73231	6-Methoxy-3-Methyl-2-All-Trans-Decaprenyl-1,4-Benzoquinol	C58H90O3	6.37E+05	0.24%
697.48527	Dioleoyl Phosphatidate	C39H71O8P	6.36E+05	0.24%
549.36921	Anhydrolutein I	C40H54O	6.26E+05	0.24%
615.06565	2-O-Galloylhyperin	C28H24O16	6.24E+05	0.24%
595.24467	Adonitoxin	C29H42O10	6.22E+05	0.24%
543.20096	3 4-Diferuloylquinic Acid	C27H28O12	6.19E+05	0.23%
315.07466	Protocatechuic Acid4-O-Glucoside	C13H16O9	6.18E+05	0.23%
209.1677	Cyclo(Pro-Leu)	C11H18N2O2	6.18E+05	0.23%
1028.23773	Stearoyl-Coa	C39H66N7O17P3S	6.11E+05	0.23%
387.24284	Medioresinol	C21H24O7	5.99E+05	0.23%
327.259	Docosahexaenoic Acid	C22H32O2	5.80E+05	0.22%
307.03041	Proline-Betaxanthin	C14H16N2O6	5.64E+05	0.21%
571.35238	Caffarolidea	C35H56O6	5.64E+05	0.21%
434.14741	Phlorizin	C21H23O10	5.63E+05	0.21%
293.05016	5-Amino-1-(5-Phospho-Beta-D-Ribosyl)Imidazole	C8H13N3O7P	5.62E+05	0.21%
291.21134	Gingerdione((6)-)	C17H24O4	5.60E+05	0.21%
336.08221	Trans-5-O-(4-Coumaroyl)-D-Quinate	C16H17O8	5.57E+05	0.21%
503.14024	17-Decarboxy-Neobetanin	C23H24N2O11	5.55E+05	0.21%
582.19008	Tricoumaroyl Spermidine	C34H37N3O6	5.53E+05	0.21%
542.20248	Trans-Zeatin-O-Glucoside-7-N-Glucoside	C22H33N5O11	5.52E+05	0.21%
449.02162	5-Phosphoribosyl-4-(N-Succinocarboxamide)-5-Aminoimidazole	C13H15N4O12P	5.46E+05	0.21%
305.08525	Convicine	C10H15N3O8	5.45E+05	0.21%
67.05447	Isoprene	C5H8	5.43E+05	0.21%
356.01473	D-Mannitol 1-Phosphate	C6H13O9P	5.30E+05	0.20%
576.39197	Tomatidine Galactoside	C33H55NO7	5.29E+05	0.20%
409.22763	2,3-Dimethyl-6-Geranylgeranyl-1,4-Benzoquinol	C28H42O2	5.29E+05	0.20%
87.04598	Allylmethylsulfide	C4H8S	5.22E+05	0.20%
571.04674	N6-(Delta2-Isopentenyl)-Adenosine 5-Triphosphate	C15H21N5O13P3	5.19E+05	0.20%
103.02713	Nicotinamide	C6H6N2O	5.19E+05	0.20%
413.24716	Lupulone	C26H38O4	5.18E+05	0.20%
456.37526	(3beta,9beta)-4alpha-Demethyl-4alpha-Carboxy-9,19-Cyclolanost-3-Ol	C30H49O3	5.17E+05	0.20%
261.15498	(2-Hydroxyphenyl)Methyl 1-	C14H14O5	5.13E+05	0.19%

	Hydroxy-6-Oxocyclohex-2-Ene-1-Carboxylate			
362.07212	Dihydrochelirubine	C21H17NO5	5.13E+05	0.19%
241.93565	Prenyl Diphosphate	C5H9O7P2	5.12E+05	0.19%
93.01553	Phenol	C6H6O	5.11E+05	0.19%
91.01585	Glycerol	C3H8O3	5.08E+05	0.19%
75.1376	Propane-1,3-Diamine	C3H12N2	5.05E+05	0.19%
567.19552	Phloretin-2-Xylosyl-Glucoside	C26H32O14	5.02E+05	0.19%
